# Arrowhead Workflow Choreography: A Formal Definition of Recipes and Their Execution Model

**DOI:** 10.3390/s26217025

**Published:** 2026-11-06

**Authors:** Pal Varga

**Affiliations:** Department of Telecommunications and Artificial Intelligence, Faculty of Electrical Engineering and Informatics, Budapest University of Technology and Economics, Műegyetem rkp. 3., H-1111 Budapest, Hungary; pvarga@tmit.bme.hu

**Keywords:** arrowhead framework, workflow, workflow choreography, service-oriented architecture, industrial Internet of Things, Industry 5.0, formal execution semantics, workflow management, system of systems, sensor networks, measurement workflows, IoT sensing

## Abstract

The Eclipse Arrowhead Framework provides a service-oriented architecture for distributed industrial automation through service discovery, authorization, orchestration, and quality-of-service-aware service selection. While the framework formally defines the execution of individual service interactions, it does not provide a mathematical semantics for coordinating multiple dependent service invocations into executable workflows. This paper presents a compact mathematical execution model for Arrowhead workflow choreography. Rather than introducing a new orchestration mechanism, the proposed model extends the Arrowhead Framework conservatively by treating workflow execution as the repeated compilation of workflow steps into ordinary Arrowhead service requests. The model introduces formal representations of workflow recipes and workflow sessions, together with a compact execution model that maps executable workflow steps to ordinary Arrowhead orchestration requests and service invocations. A worked example demonstrates how the notation can be instantiated for a generic setting, whereas another example shows a concrete temperature-control workflow, and a third example a shared-tool delivery workflow in a forklift assembly plant. The resulting formalization preserves the service-oriented semantics of the Arrowhead Framework while providing a mathematically precise model for workflow execution, including measurement workflows that coordinate sensing, validation and actuation services, in dynamic industrial environments.

## 1. Introduction

Industrial automation is in the middle of a transition from the flexible, service-oriented paradigm of Industry 4.0 [1] towards the human-centric, resilient, and sustainability-driven objectives of Industry 5.0 [2,3]. This shift raises the bar for the runtime flexibility of production systems: complex tasks can no longer be fixed at design time but must be composed, reconfigured, and executed dynamically across heterogeneous devices, systems, and organizational boundaries [4,5].

The Eclipse Arrowhead Framework has emerged as the most widely adopted realization of the service-oriented architecture paradigm [6] for Industrial Internet of Things (IIoT), cyber–physical production systems (CPPSs) [7] and Industry 4.0/5.0 System-of-Systems (SoS) engineering [8,9]. Its core architecture provides standardized mechanisms for service discovery, authorization, orchestration, quality-of-service (QoS) management, and secure inter-cloud communication, thereby enabling interoperability among heterogeneous cyber–physical systems [10]—a goal shared by other Industrial IoT interoperability standards and reference architectures, such as OPC UA and RAMI 4.0 [11,12].

The formal definitions of the Arrowhead core systems, recalled in Section 3, characterize their behavior in detail. They describe how application systems publish services, how consumers formulate service requests, and how the Orchestrator dynamically selects service providers while respecting authorization and QoS constraints. Consequently, the Arrowhead Framework already provides a rigorous mathematical model for the execution of individual service interactions.

### Motivation

Individual, isolated service invocations are, however, rarely sufficient to realize a real production task. Practical Industry 4.0/5.0 applications typically require several dependent service interactions to be coordinated into workflows—for instance, in digital production, product-lifecycle management, and supply-chain scenarios. Recognizing this need, prior work introduced a dynamic, multilevel workflow management concept for Arrowhead-based IIoT systems [13], data-driven workflow execution based on BPMN and colored Petri nets [14], and, most directly, the Arrowhead workflow choreographer, which enables flexible digital production by dynamically composing ordinary Arrowhead service requests into executable workflows [15]. Complementary microservice-based workflow-management solutions have also been investigated for industrial automation [16], and the wider challenge of engineering and lifecycle-managing such large-scale, complex SoS ecosystems has been studied from a process-engineering perspective [17]. Many of these workflows are centered on sensing. In a typical measurement workflow, sensor values are acquired, validated, for example, against redundant sensors or calibration status, combined into a measurement result, and finally used for control or actuation. In an Arrowhead Local Cloud, sensors, evaluation functions and actuators are all services, so coordinating such a measurement workflow is a coordination of dependent service interactions. The present paper does not propose a new measurement method. It formalizes how the services that perform measurements are coordinated, and thereby states precisely, for example, that a step consuming a measurement can start only after the steps that produce it have completed (Lemma 1). The worked examples of Section 7 and Section 8 illustrate this on a temperature measurement-and-control workflow and on a tightening workflow whose measurement results are evaluated and recorded. Despite this substantial body of architectural and practical work, none of these contributions provide mathematically precise execution semantics for workflow choreography that is directly grounded in—and conservatively compatible with—the formal Arrowhead model recalled in Section 3. Existing publications describe *what* the choreographer does and *how* it is architected and deployed, but not how its coordination of steps, actions, and plans can be expressed as a compact, composable mathematical object consistent with the orchestration function already defined for individual service interactions. This gap is not merely academic: without a formal execution model, properties such as well-formedness of a workflow, determinism of step scheduling, or the precise boundary between what workflow choreography decides and what the Arrowhead Orchestrator decides cannot be stated, let alone verified. This paper closes that gap.

Although the Eclipse Arrowhead project provides workflow choreography as a practical mechanism for coordinating dependent service interactions, its operational behavior has therefore not yet been described through a unified mathematical model. The objective of this paper is to extend the Arrowhead Framework’s formalism with such a model. Unlike conventional workflow engines, workflow choreography does not perform service discovery or provider selection itself. Instead, each workflow step is dynamically transformed into an ordinary Arrowhead service request and executed through the orchestration process already defined by the Arrowhead Framework. Workflow choreography therefore contributes only the coordination semantics required for executing workflows, while preserving the existing Arrowhead service-oriented architecture.

The main contributions of this paper are the following.

(i) A mathematical representation of workflow recipes;
(ii) A workflow session model for runtime execution;
(iii) A compact execution model showing how steps are compiled into Arrowhead requests and executed through Arrowhead orchestration;
(iv) A summary of the guarantees of the model that are relevant for engineers (Section 5.11): static validation of recipes, data-availability safety, bounded runs, and exclusive use of shared resources;
(v) Worked examples demonstrating the model on concrete workflows, including a shared-tool delivery workflow of a forklift assembly plant.

Taken together, these contributions establish workflow choreography as a conservative mathematical extension of the Arrowhead Framework. Every service invocation remains governed by the Arrowhead core systems, whereas workflow choreography determines only the ordering and coordination of those invocations.

## 2. Related Work

### 2.1. Service-Oriented Architectures for Industrial IoT

Service-oriented architecture (SOA) has long been established as a design paradigm for building loosely coupled, interoperable systems from independently deployed services [6]. Its adoption in industrial automation has been driven by the need to integrate heterogeneous devices, controllers, and enterprise systems without imposing a single vendor-specific protocol; interoperability standards such as OPC UA compete with and complement lighter-weight IoT protocols such as MQTT and DDS in this space, each offering different trade-offs between semantic richness and runtime performance [11]. At the architectural level, the Reference Architecture Model Industrie 4.0 (RAMI 4.0) provides a three-dimensional frame for positioning such interoperability layers within the broader lifecycle and hierarchy of an industrial asset, and has itself been used as the basis for concrete service-discovery architectures in manufacturing [12]. The Eclipse Arrowhead Framework is one realization of this SOA vision specifically for Industrial IoT: it structures interoperability around Local Clouds containing mandatory core systems for Service Registry, orchestration, and authorization [8,9]. Unlike RAMI 4.0, which is a reference model rather than a deployable architecture, Arrowhead provides running core systems and a documented service-request lifecycle, which is what makes a mathematical execution model for it meaningful and directly implementable.

**Figure 1:**
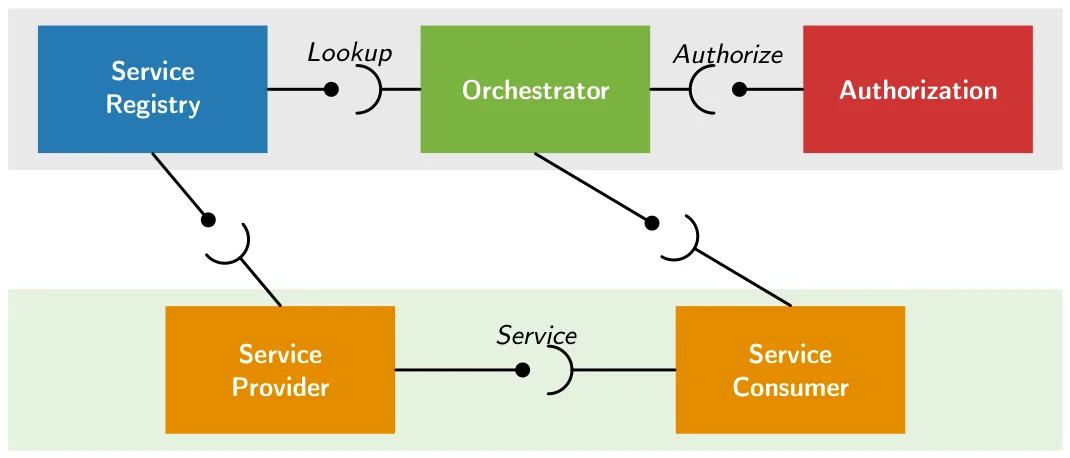
The simplest architectural diagram for an Eclipse Arrowhead Local Cloud—with core systems on the top, and two application systems on the bottom.

A minimalistic Local Cloud setup is illustrated in Figure 1 The connectors suggest the loose coupling, late binding features of the service-oriented architecture that serves as a principle concept for Eclipse Arrowhead. All of these connections are seen as a given service, which has a consumer and a provider. There are services such as Lookup or Authorize, as shown.

This architectural line of work is further motivated by the ongoing shift from Industry 4.0’s efficiency-and-connectivity focus towards the human-centric, resilient, and sustainability-oriented objectives of Industry 5.0 [2,3]. Both perspectives agree that the runtime flexibility of production systems—their ability to reconfigure and recompose services on demand—is a prerequisite for meeting these objectives, which is precisely the capability that workflow choreography adds on top of the Arrowhead core.

Arrowhead has also been applied to sensor integration in semiconductor manufacturing [18] and to smart city and smart agriculture solutions [19]. These works use the Arrowhead core systems for discovery, authorization and orchestration of individual services, and do not address the coordination of dependent services in a workflow.

### 2.2. Formal Models of Service Orchestration and Choreography

Outside the Arrowhead ecosystem, orchestration and choreography have been studied extensively as two complementary views of multi-service coordination: orchestration describes coordination from the perspective of a single controlling party, whereas choreography describes the externally observable, peer-to-peer interaction contract among several parties [20]. This distinction motivated standardization efforts such as the Web Services Choreography Description Language (WS-CDL), for which formal models and verification procedures were proposed to give its interaction contracts precise, checkable semantics [21]. On the orchestration side, process algebras have been used to give languages such as WS-BPEL compositional semantics: π-calculus-based semantics for WS-BPEL, for instance, makes properties such as deadlock-freedom and behavioral equivalence of orchestrated processes amenable to formal reasoning [22]. A parallel and older tradition formalizes workflow coordination using Petri nets, which provide both an intuitive graphical notation and a rich analysis toolbox for properties such as soundness and termination of a workflow [23]; the workflow-patterns framework built on this tradition to catalog the control-flow, data, and resource patterns that any sufficiently expressive workflow language must support [24]. More recently, this line of work has been extended to give the de facto industry notation, BPMN, a precise operational semantics amenable to automated analysis [25].

These formalisms target general-purpose business-process or web-service coordination, in which the executing services are either statically bound or resolved through registries external to the coordination model itself. Workflow choreography, in contrast, coordinates steps whose providers are resolved dynamically, at each execution, by the Arrowhead Orchestrator—so a workflow recipe never binds to a concrete provider and remains valid as the population of available services changes. None of the formalisms above are defined against such a dynamically orchestrated service substrate, which is the specific technical gap that this paper addresses.

Timed models of distributed systems have also been used to check deadlock freedom and termination automatically [26]. The present paper establishes these properties for workflow sessions by proof (Theorems 1 and 2).

### 2.3. Foundations and Surveys of Workflow Management

The formal model developed in this paper sits on top of a large, mature body of work on workflow management as a discipline in its own right. workflow management emerged as a distinct research area once organizations began separating the specification of a business process from the applications that carry out its individual steps. The foundational survey by Georgakopoulos, Hornick, and Sheth traces this separation from early process-modeling notations through to workflow automation infrastructures, and already identifies the recurring architectural components—a process model, a workflow engine, and a set of participating applications or agents—that essentially every later workflow-management system, including Arrowhead’s choreographer, still instantiates in some form [27]. Business process management (BPM) subsequently broadened this scope beyond automation to cover the full life cycle of a process—design, configuration, enactment, and diagnosis—and van der Aalst’s comprehensive survey of the field organizes several decades of BPM research along exactly these life-cycle phases, while also cataloging the process-modeling notations (Petri nets, BPMN, YAWL, and others) that a workflow engine may be asked to execute [28]. These two surveys jointly establish the vocabulary—process/workflow model, case, task, resource, engine—against which more specialized workflow-management literature, including the manufacturing- and supply-chain-specific work discussed next, can be positioned.

### 2.4. Workflow Management for Manufacturing

In manufacturing, workflow management has increasingly been studied under the heading of workflow scheduling: given a process model whose tasks are annotated with resource and timing requirements, decide which task runs where and when. A recent survey specifically targeting Industry 4.0 settings categorizes industrial workflow-scheduling approaches by objective (makespan, cost, energy, reliability), by solution technique (heuristic, metaheuristic, learning-based), and by the degree of dynamism assumed in the shop-floor environment [29]. A closely related line of work addresses workflow scheduling in cloud manufacturing, where manufacturing capabilities themselves are exposed as on-demand, elastic services; Liu et al. survey the resulting scheduling problem across service composition, task allocation, and quality-of-service dimensions and identify the lack of unified, dynamically re-schedulable execution models as an open challenge [30], while Rashidifar, Bouzary, and Chen focus specifically on resource-scheduling strategies for cloud-based manufacturing systems and compare centralized against decentralized scheduling architectures [31]. Across all three surveys, workflow scheduling is treated primarily as an optimization problem layered on top of an already-executable process model; none of them provide, or presuppose, formal execution semantics for how an individual scheduled task is compiled into a concrete service invocation, which is the concern that this paper addresses for the Arrowhead case.

Within the Arrowhead ecosystem specifically, digital-production workflow management has been surveyed and developed as part of the System-of-Systems engineering agenda around the framework. An early survey positions Arrowhead-based service orchestration against the broader landscape of digital production, product-lifecycle, and supply-chain management solutions for Industry 4.0, identifying dynamic workflow coordination as a capability that the base framework lacked at the time [14]. The Arrowhead workflow choreographer was subsequently introduced to close exactly that gap, enabling flexible digital production through dynamic composition of Arrowhead service requests [15], and was later generalized into a multilevel workflow-management concept spanning individual production steps up to plant-wide coordination [13]. This paper’s formal model targets precisely the execution layer of that choreographer, complementing its engineering description with a mathematical one.

Workflow orchestration has also been studied for sensor-driven processes: BPMN has been extended with sensor and actuator tasks [32,33], and distributed workflow applications have been orchestrated across cloud, edge and IoT devices [34,35].

Exception handling and adaptive adjustment of service compositions under time constraints in cloud manufacturing are treated in [36]; in our model, failure handling is limited to the rules S-FailBind and S-FailInvoke.

The execution semantics of Arrowhead workflow choreography is not the subject of these works.

### 2.5. Workflow Management for Supply Chains

At the supply-chain level, workflow management concerns the coordination of processes that cross organizational boundaries—procurement, production planning, logistics, and delivery—rather than tasks within a single shop floor. Ben-Daya, Hassini, and Bahroun’s literature review of IoT and supply-chain management shows how real-time sensor and traceability data increasingly drives supply-chain process execution, and explicitly calls for architectures that can translate such data into automatically triggered process steps rather than manually configured ones [37]. Frederico, Garza-Reyes, Anosike, and Kumar’s review of “Supply Chain 4.0” surveys the maturity of digital supply-chain concepts and proposes a research agenda in which process orchestration across tiers of suppliers is identified as a key open capability [38], while Zhang, Yang, and Yang’s more recent review of smart supply-chain management under Industry 4.0 surveys the specific technologies—IoT platforms, digital twins, and cloud-based coordination services—through which such orchestration has so far been attempted in practice [39]. A complementary strand of work studies blockchain-based coordination as an alternative to centralized workflow engines for cross-organizational supply-chain processes; Wannenwetsch et al. review this literature and note that most blockchain-based proposals focus on trust and traceability guarantees rather than on precise execution semantics for the underlying process steps themselves [40].

Within the Arrowhead ecosystem, supply-chain coordination has been studied as a direct application area for the framework’s service-oriented mechanisms. Kozma, Varga, and Hegedűs examine how Arrowhead’s Local Cloud and inter-cloud mechanisms can support supply chain management and Logistics 4.0 scenarios, integrating discrete logistics events into the same orchestration model used for shop-floor services [41]. As in the manufacturing case, this line of work motivates that Arrowhead-based workflow coordination is applicable to supply-chain processes, but stops short of giving the underlying coordination mechanism—the choreographer—a formal execution model.

### 2.6. Summary of Workflow Management Within the Arrowhead Framework

Altogether, within the Arrowhead ecosystem itself, the need for workflow-level coordination on top of individual service interactions has been recognized and addressed at the architectural and engineering level. The Arrowhead workflow choreographer was introduced as a mechanism for achieving flexible digital production by dynamically composing Arrowhead service requests into executable process chains [15], and was subsequently embedded into a broader, multilevel workflow-management concept spanning individual production steps up to plant-wide coordination [13]. A complementary line of work investigated data-driven workflow execution by combining Arrowhead with established process-modeling notations, representing workflows as BPMN diagrams that are compiled into executable colored Petri nets [14], and microservice-based workflow management solutions have been evaluated as an alternative implementation strategy for the same coordination problem [16]. This body of work is also situated within the wider Industry 5.0 research agenda pursued around the Arrowhead Framework, which highlights AI adoption, human acceptance, and engineering methodology as open challenges for SoS-based production systems.

### 2.7. Positioning of the Present Work

Table 1 summarizes the five streams of related work surveyed in Section 2.2, Section 2.3, Section 2.4, Section 2.5 and Section 2.6, condensing each stream’s representative works, its coordination focus, whether it provides a formal execution semantics, and the specific gap that remains open relative to the model developed in this paper. Read top to bottom, the table traces a consistent pattern: formal semantics exist, but only for statically bound or externally resolved coordination languages (row 1); Arrowhead-specific workflow coordination exists, but only operationally (row 2); and the manufacturing- and supply-chain-facing literature treats scheduling, IoT integration, and blockchain trust as largely independent concerns layered above an already-executable process model (rows 3–5). None of the five streams formalize the compilation of a workflow task into a dynamically orchestrated service request—the specific layer that this paper’s workflow recipe and workflow session model supplies.

Taken together, the workflow-management literature surveyed above establishes two things relevant to this paper. First, workflow scheduling and coordination for both manufacturing and supply-chain processes is an active and well-studied problem, addressed from optimization, IoT-integration, and blockchain-coordination perspectives [29,30,31,37,38,39,40]. Second, none of these surveys—general, manufacturing-specific, or supply-chain-specific—treat the compilation of a workflow task into a concrete, dynamically orchestrated service request as a formal object in its own right; scheduling and coordination are studied above an assumed-executable process model, not at the level of what makes a single step executable to begin with. The Arrowhead-based studies reviewed here come closest to the present paper’s setting, since they operate directly on top of the same service-oriented core [13,15,41], yet they too describe workflow coordination operationally rather than mathematically. The workflow recipe and workflow session model introduced in this paper is intended to supply exactly that missing formal layer, applicable without modification to both the manufacturing and supply-chain workflow scenarios surveyed above, since both ultimately reduce, in the Arrowhead setting, to a sequence of steps compiled into orchestration requests.

Altogether, prior work establishes that Arrowhead-based workflow coordination is architecturally feasible and practically useful, and explores several concrete implementation strategies for it—BPMN/CPN compilation, microservice composition, and the choreographer itself. What none of it provides is a mathematical execution semantics that ties this coordination layer back to the underlying Arrowhead orchestration function in a conservative, verifiable way. The workflow recipe and workflow session model introduced in this paper is intended to fill exactly that gap, complementing the engineering contributions above with a formal counterpart.

## 3. Mathematical Context

Throughout this paper, the mathematical model of the Arrowhead Framework’s core systems and services, as established by the published Arrowhead architecture [8,9,10], is assumed. Only those concepts required for the workflow formalization are recalled here.

Workflow choreography introduces no alternative service discovery, authorization, or orchestration mechanisms. Instead, every workflow step is transformed into an ordinary Arrowhead service request and executed according to the semantics of the Arrowhead core systems recalled above.

### 3.1. The Brief Formal Definition of the Main Eclipse Arrowhead Concepts

Let $LC_{i}$ denote an Arrowhead Local Cloud.

A Local Cloud is modeled as a static structure together with an explicitly time-dependent local state. Its static structure in its briefest form is

$${\mathrm{LC}}_{i}=\left({\mathrm{APP}}_{i},C_{i}^{min},C_{i}^{sup},\sigma_{i}\right),$$

where:

- ${\mathrm{APP}}_{i}$ is the set of application systems in Local Cloud *i*.
- $C_{i}^{min}$ is the set of mandatory core systems.
- $C_{i}^{sup}$ is the set of supporting core systems considered in this model.
- $\sigma_{i}$ is the set of abstract service types known in Local Cloud *i*.

The mandatory core systems are $C_{i}^{min}=\left\{R_{i},O_{i},A_{i}\right\}$, where $R_{i}$ denotes the Service Registry, $O_{i}$ denotes the Orchestrator, and $A_{i}$ denotes the authorization system.

There are many supporting core systems in the Arrowhead framework; a basic subset is $\left\{GK_{i},GW_{i},QM_{i}\right\}\subseteq C_{i}^{sup}$, where $GK_{i}$ denotes the gatekeeper, $GW_{i}$ denotes the gateway, and $QM_{i}$ denotes the QoS manager. The gateway, gatekeeper, and QoS manager are modeled as supporting systems, not as mandatory members of every Local Cloud. The latter will not be utilized for the current concept of workflow choreography.

The set of all systems inside Local Cloud *i* is ${\mathrm{SYS}}_{i}={\mathrm{APP}}_{i}\cup C_{i}^{min}\cup C_{i}^{sup}$.

When introducing time dependency, the Service Registry is represented by $R_{i}\left(t\right)$, where t∈T denotes system time.

Service requests belong to the request space $Req_{i}$.

The Arrowhead Orchestrator is represented by the orchestration function $O_{i}:Cons_{i}\times Req_{i}\times \Gamma_{i}\times T⟶\Pi_{i}\cup \left\{⊥\right\}$, where $Cons_{i}$ is the set of systems that may act as service consumers, $Req_{i}$ is the orchestration request, and Γ is the orchestration-constraint space at a given time *T*—hence, $O_{i}$ either returns a feasible orchestration plan $\pi \in \Pi_{i}$, or the failure symbol ⊥.

Authorization, quality-of-service filtering, gateway communication, and gatekeeper functionality follow their standard Arrowhead Framework definitions [8,9] and are not modified here; they therefore remain outside the scope of the present formalization.

The workflow semantics developed in the remainder of this paper interacts with the Arrowhead Framework exclusively through the orchestration function $O_{i}$.

Table 2 summarizes the symbols used for the Arrowhead core systems and service requests recalled in this section.

### 3.2. Scope of the Workflow Choreography

Workflow choreography introduces three new mathematical concepts.

1. The *Workflow Recipe*, describing the static dependency structure of workflow execution.
2. The *Workflow Session*, representing one runtime execution of a workflow recipe.
3. A lightweight execution model describing how workflow sessions evolve during normal workflow execution.

The relationship between the Arrowhead Framework and workflow choreography is summarized by

- *Workflow Recipe*⟹
- ⟹*Workflow Session*⟹
- ⟹*Arrowhead Requests*⟹
- ⟹*Arrowhead Orchestration*⟹
- ⟹*Workflow State Evolution*

This hierarchy forms the basis of the mathematical model developed in the following sections.

## 4. Workflow Recipe

The workflow recipe is the fundamental static object of workflow choreography. It specifies the logical execution structure of an application independently of the current state of the Arrowhead Framework.

Unlike executable workflow instances, the workflow recipe contains no runtime information. In particular, it does not specify concrete service providers, orchestration plans, execution states, or intermediate execution results.

Instead, the workflow recipe defines

- The hierarchical organization of the workflow;
- The dependency relations between workflow activities;
- The service requirements associated with each activity.

During execution, the workflow recipe remains immutable. Every runtime property is maintained separately by the workflow session introduced in Section 5.

### 4.1. Hierarchical Structure

A workflow recipe consists of three hierarchical levels:

$$\begin{array}{c} Plan⊃Actions⊃Steps \end{array}$$

The plan represents the complete workflow.

Each plan consists of a finite collection of actions.

Each action contains a finite directed acyclic graph of steps.

Only steps interact directly with the Arrowhead Framework.

### 4.2. Plan

Every workflow recipe contains exactly one plan *P*.

The plan is defined as $P=(name,A,\alpha_{0},next_{A})$.

The components are
     
name
workflow identifier     
*A*
finite action set     
$\alpha_{0}$
initial action     
${\mathrm{next}}_{A}$
action transition relation

The finite action set is

$$A=\{\alpha_{0},\alpha_{1},\ldots ,\alpha_{n}\}.$$

The initial action satisfies $\alpha_{0}\in A$.

### 4.3. Action Graph

The execution order between actions is represented by $next_{A}\subseteq A\times A$.

Consequently, the plan induces the directed graph $G_{A}=\left(A,E_{A}\right)$, where $E_{A}=next_{A}$.

The action graph satisfies the following properties.

**Definition** **1** (Action Graph)**.** *The action graph is*

*1.*  *Finite;*
*2.*  *Acyclic;*
*3.*  *Weakly connected.*

Unlike workflow steps, actions execute sequentially.

Therefore, outdeg(α)≤1 for every action. Moreover, every action is reachable from the initial action $\alpha_{0}$ in $G_{A}$. Together with outdeg(α)≤1 and acyclicity, this means that $G_{A}$ is a single directed path $\alpha_{0}\to \alpha_{1}\to \cdots \to \alpha_{n}$, and that $\alpha_{0}$ has no incoming edge.

### 4.4. Actions

Each action represents one logical execution stage.

An action consists of $\alpha =(S_{\alpha },F_{\alpha },next_{\alpha })$.

The step set is $S_{\alpha }=\left\{s_{1},\ldots ,s_{m}\right\}$.

The step sets of distinct actions are pairwise disjoint, $S_{\alpha }\cap S_{\beta }=⌀$ for α≠β. Hence, every step s∈S belongs to exactly one action, denoted α(s).

The subset $F_{\alpha }=\left\{\;s\in S_{\alpha }|pre\left(s\right)=⌀\;\right\}\subseteq S_{\alpha }$ is *exactly* the set of steps without predecessors, where pre(s) is derived from $next_{\alpha }$ as defined in the subsection on Step Functions.

### 4.5. Step Graph

Execution dependencies inside one action are represented by $next_{\alpha }\subseteq S_{\alpha }\times S_{\alpha }$.

The corresponding graph is $G_{\alpha }=\left(S_{\alpha },E_{\alpha }\right)$, where $E_{\alpha }=next_{\alpha }$.

**Definition** **2** (Step Graph)**.** *For every action, $G_{\alpha }$ is*

*1.*  *Finite;*
*2.*  *Acyclic.*

*Consequently, parallel execution is permitted whenever multiple successor steps become enabled simultaneously.*

### 4.6. Steps

The complete step universe of the workflow recipe is $S={⋃}_{\alpha \in A}S_{\alpha }$.

Each step represents one logical service interaction.

Unlike classical workflow languages, a step specifies only service requirements. Concrete service providers are determined dynamically during runtime by the Arrowhead Orchestrator.

Each step s∈S is an identifier.

Its requirement profile is the tuple $\sigma \left(s\right)=\left(\theta (s),\iota (s),\mu (s),q(s),pre(s),\eta (s),qnty(s)\right)$, whose components are the functions on *S* defined in the next subsection.

The individual components of the profile are summarized below.
θrequired service definitionιrequired interfaceμmetadata constraints*q*QoS requirementsprepredecessor step setηoptional workflow executor capabilityqntyexecution multiplicity

### 4.7. Step Functions

Rather than storing information procedurally, each component of a step is represented by a mathematical function.

The service definition is θ:S→Θ.

The required interface is ι:S→I.

Metadata requirements are μ:S→MD.

Quality-of-service requirements are q:S→Q.

Predecessor relations are $pre:S\to 2^{S}$. The function pre is not an independent component: it is derived from the step-dependency relations of the actions, $pre\left(s\right)=\{\;p\in S_{\alpha (s)}|\left(p,s\right)\in next_{\alpha (s)}\;\}$.

Conversely, $next_{\alpha }=\left\{\left(p,s\right)|s\in S_{\alpha },\;p\in pre\left(s\right)\right\}$, so the two descriptions are interchangeable.

Workflow executor capabilities are η:S⇀E, where undefined values indicate direct execution by the workflow choreographer.

Finally, execution multiplicity is qnty:S→N.

### 4.8. Data Dependencies

The predecessor function pre is a *control* dependency: it determines *when* a step may start. Data dependencies determine *what* a step receives, and are specified separately.

Let *D* be the application-specific data domain and P the domain of *session parameters*, i.e., constants (thresholds, set points, request templates) that are supplied when a workflow session is created and stay fixed during the session. For a finite set U⊆S, let $D^{U}$ denote the set of maps U→D. For every step s∈S, the recipe fixes $dep:S\to 2^{S},\;\;\kappa_{s}:P\times D^{dep(s)}\to D$, where dep(s) is the set of steps whose results *s* consumes, and $\kappa_{s}$ is the *input-composition function* of *s*: it builds the payload passed to the selected service from the session parameters and from the results of the steps in dep(s). Since $\kappa_{s}$ takes a map indexed by steps and not a sequence, its value does not depend on the order in which parallel predecessors complete, so the input of a join step is deterministic.

Let $pre^{+}$ be the transitive closure of pre, and write $\alpha \left(p\right)\to^{+}\alpha \left(s\right)$ if α(s) is reachable from α(p) by at least one edge of $G_{A}$. A step *p precedes* a step *s*, written p≺s, if $p\in pre^{+}\left(s\right)$ (same action) or $\alpha \left(p\right)\to^{+}\alpha \left(s\right)$ (earlier action). A workflow recipe is well-formed only if every step consumes results that are guaranteed to exist when the step is enabled: dep(s)⊆{p∈S∣p≺s}foralls∈S (Lemma 2). Data may therefore also flow between actions, because all steps of an earlier action are completed before any step of a later action is enabled (Lemma 1(iv)). If $\kappa_{s}$ is specified by a template that refers to results by step identifier, then dep(s) is exactly the set of steps referred to by the template. Hence, dep is inferred from the input specifications, and the condition above can be checked statically on the recipe, before any session is started.

### 4.9. Workflow Recipe

Collecting the previous definitions, a workflow recipe is represented by W=(P,A,S,θ,ι,μ,q,pre,η,qnty,dep,κ).

**Figure 2:**
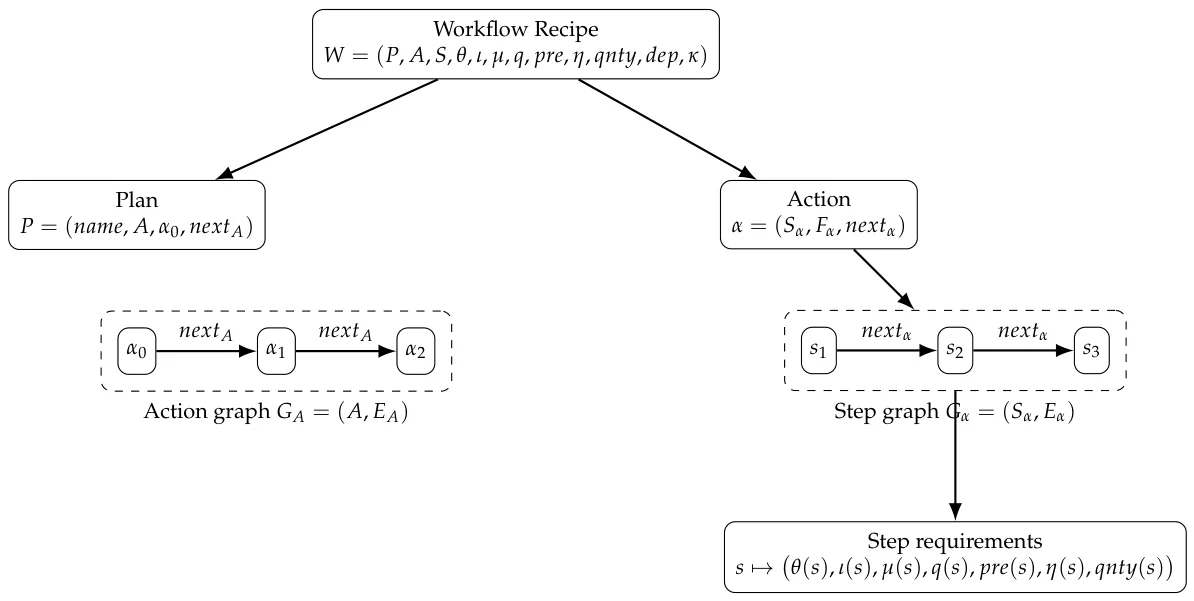
Hierarchical structure of a workflow recipe. The plan defines the action-level control structure, each action contains an internal step graph, and each step carries the service and execution requirements used later by the workflow execution model.

Figure 2 visualizes this by showing a hierarchical recipe diagram.

Well-formedness.

A workflow recipe *W* is *well-formed* if

(W1) *A* is finite, $G_{A}$ is acyclic with outdeg≤1, and every action is reachable from $\alpha_{0}$ (hence $G_{A}$ is a directed path from $\alpha_{0}$);
(W2) The step sets $S_{\alpha }$ are finite and pairwise disjoint, and every $G_{\alpha }$ is acyclic;
(W3) pre is derived from $next_{\alpha }$ and $F_{\alpha }=\left\{s\in S_{\alpha }|pre\left(s\right)=⌀\right\}$;
(W4) dep(s)⊆{p∈S∣p≺s} for every s∈S.

All results of Section 5 assume well-formed recipes.

Notice that neither execution state, service bindings, nor execution results belong to the workflow recipe.

Consequently, workflow recipes remain immutable throughout workflow execution.

Every runtime property introduced later is maintained exclusively by workflow sessions.

Table 3 summarizes the symbols used to define the workflow recipe in this section.

## 5. Workflow Execution Model

The workflow recipe specifies the static structure and service requirements of a workflow. It does not contain execution states, concrete service providers, or intermediate results. These elements arise only when the recipe is instantiated and executed.

Workflow execution is realized by repeatedly transforming executable steps into ordinary Arrowhead service requests. Each generated request is submitted to the Arrowhead Orchestrator, which determines a feasible service provider according to the Service Registry, authorization rules, interface constraints, and quality-of-service requirements captured by the orchestration function $O_{i}$ recalled in Section 3.

Workflow choreography therefore introduces no alternative service-discovery or provider-selection mechanism. Its responsibility is limited to

(i) Determining which steps may execute;
(ii) Compiling those steps into Arrowhead requests;
(iii) Initiating the resulting service interactions;
(iv) Updating the runtime state of the workflow.

### 5.1. Execution Pipeline

Let W=(P,A,S,θ,ι,μ,q,pre,η,qnty,dep,κ) be a well-formed workflow recipe.

The execution of a step s∈S follows the conceptual pipeline finishing with the workflow state update

$$\begin{array}{c} \mathrm{Step}\;\overset{\mathrm{compile}}{\to }\;\mathrm{Arrowhead}\;\mathrm{Req}.\;\overset{O_{i}}{\to }\;\mathrm{Orch}.\;\mathrm{Plan}\;\overset{\mathrm{consume}}{\to }\;\mathrm{Exec}.\;\mathrm{Result}\;⟹\;\mathrm{State}\;\mathrm{Upd}. \end{array}$$

The first transformation is defined by workflow choreography. Provider selection is performed exclusively by the Arrowhead orchestration function $O_{i}$. Service invocation is represented abstractly so that the model remains independent of concrete communication protocols and implementation technologies.

### 5.2. Workflow Choreographer and Workflow Executors

Let $CH_{i}\in Cons_{i}$ denote the workflow choreographer operating in Local Cloud $LC_{i}$.

Some steps may be invoked directly by $CH_{i}$. Other steps may be delegated to dedicated workflow executors. Let $WE_{i}\subseteq Cons_{i}$ denote the finite set of workflow executors available to the choreography system.

The function η:S⇀E belongs to the workflow recipe and specifies an optional executor capability required by a step, where *E* is the set of workflow executor capability descriptions. If η(s) is undefined, step *s* is executed directly by the workflow choreographer. If η(s) is defined, a compatible workflow executor must be assigned at runtime.

The runtime executor assignment is represented by the partial function $\varepsilon :S⇀WE_{i}$.

Capabilities and satisfaction.

Each workflow executor $w\in WE_{i}$ has a finite *capability set* cap(w)⊆E, that is, $cap:WE_{i}\to 2^{E}$. The capability set is fixed when *w* is registered at $CH_{i}$ and does not change during a workflow session. The *satisfaction relation* between an executor $w\in WE_{i}$ and a capability description e∈E is

w⊧e⟺e∈cap(w).

A step *s* with η(s) defined can therefore be executed exactly by the executors of the candidate set $\left\{\;w\in WE_{i}|w⊧\eta \left(s\right)\;\right\}$. If this set is empty, the step cannot be bound.

Valid assignment.

Let *z* be a workflow session state with executor assignment ε. The assignment ε is *valid* (for the recipe *W*) if

(V1) dom(ε)⊆dom(η): only steps that require a capability have an assigned executor;
(V2) ε(s)⊧η(s) for every s∈dom(ε): the assigned executor has the required capability;
(V3) ε(s) is defined for every step *s* with η(s) defined and M(s)∈{running,completed}: a step that has been bound or has completed has an executor.

The assignment $\varepsilon_{0}$ of the initial state is empty and valid. The transition rules preserve validity (Lemma 1(v)), because only S-Bind changes ε, and only through asg(z,s). If η(s) is undefined, step *s* is executed directly by the workflow choreographer.

For a workflow session *z*, the consumer responsible for step *s* is

$$c_{z}\left(s\right)=\left\{\begin{array}{cc} CH_{i}, & \eta (s)\;\mathrm{is}\;\mathrm{undefined},\\ \varepsilon_{z}\left(s\right), & \eta (s)\;\mathrm{is}\;\mathrm{defined}. \end{array}\right.$$

Consequently, $c_{z}\left(s\right)$ is defined for every step *s* with η(s) undefined, and for every step with η(s) defined and $\varepsilon_{z}\left(s\right)$ defined, in particular, by (V3), for every running or completed step. The executor is chosen at the transition S-Bind, before the Orchestrator is called with $c_{z}\left(s\right)$ as consumer.

### 5.3. Workflow Session State

A workflow session is one runtime execution of a workflow recipe *W* inside Local Cloud $LC_{i}$. Its state is represented by $z=\left(M,M_{A},\beta ,\varepsilon ,\xi ,status\right)\in Z_{i}$.

The step-marking function is M:S→{inactive,enabled,running,completed}, and the action-marking function is $M_{A}:A\to \left\{\mathrm{inactive},\mathrm{active},\mathrm{completed}\right\}$.

The binding function is $\beta :S\to \Pi_{i}\cup \left\{⊥\right\}$, where β(s)=⊥ means that step *s* currently has no service binding.

The runtime executor assignment is $\varepsilon :S⇀WE_{i}$, and the execution context is $\xi :S⇀X_{i}$, where $X_{i}$ denotes the domain of intermediate step-local execution data, including service results and data needed by successor steps. In this model, $X_{i}=D$: the context ξ stores the values returned by $consume_{i}$ (Section 5.7), so that they can be used by the input-composition functions $\kappa_{s}$ of successor steps (Section 4.8).

Finally, status∈{running,completed,failed} records the overall status of the workflow session.

Initial state.

A workflow session of a well-formed recipe *W* with session parameters ρ∈P starts in the initial state $z_{0}=\left(M_{0},M_{A,0},\beta_{0},\varepsilon_{0},\xi_{0},status_{0}\right)$, with

$$M_{0}\left(s\right)=\mathrm{inactive},\;\beta_{0}\left(s\right)=⊥,\;\varepsilon_{0}\left(s\right)\;\mathrm{and}\;\xi_{0}\left(s\right)\;\mathrm{undefined}\;\left(s\in S\right),$$

$$M_{A,0}\left(\alpha_{0}\right)=\mathrm{active},\;M_{A,0}\left(\alpha \right)=\mathrm{inactive}\;\;\left(\alpha \neq \alpha_{0}\right),\;status_{0}=\mathrm{running}.$$

**Figure 3:**
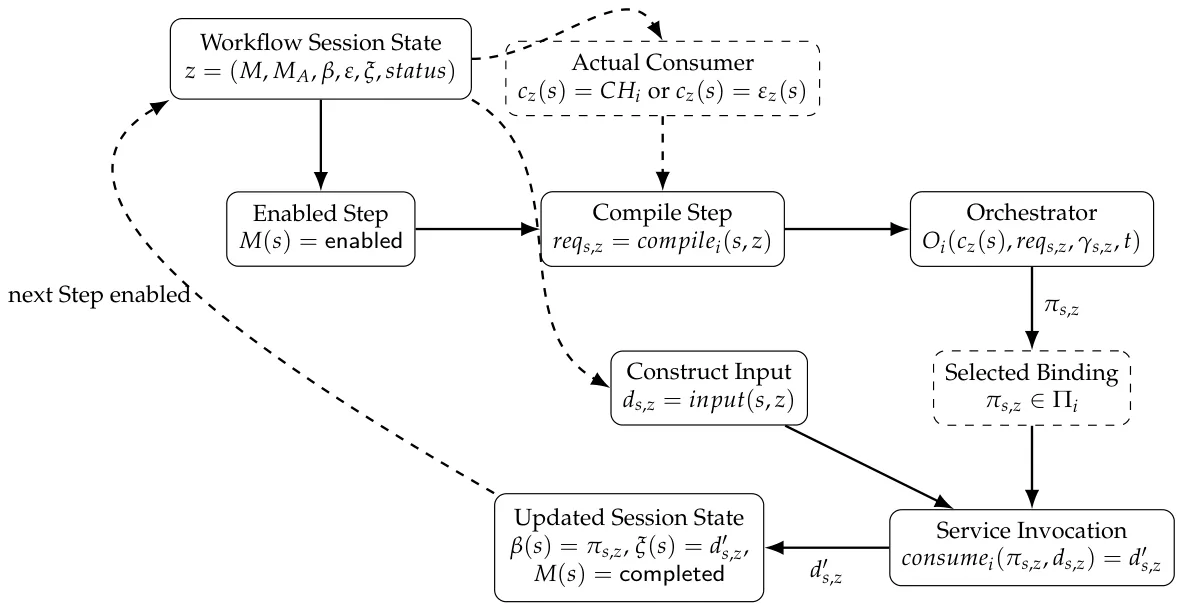
Workflow execution model for one enabled step. The current workflow session state determines the enabled step, the actual consumer, and the input data. The step is compiled into an Arrowhead orchestration request, the Orchestrator selects a service binding, the selected service is invoked, and the result is stored back into the session state.

The parameters ρ are constant throughout the session and are therefore not a component of *z*. Figure 3 shows the execution model for one enabled step, starting from a given workflow session state.

### 5.4. Step Input Data

Let *D* denote the application-specific data domain.

The data supplied to a step is composed from the session parameters and from the results of the steps that it depends on, as specified by the recipe through dep and $\kappa_{s}$ (Section 4.8). For a step *s* in a session *z*, $input\left(s,z\right)\;=\;\kappa_{s}\left(\rho ,\;\xi_{z}{|}_{dep(s)}\right)$, where $\xi_{z}{|}_{dep(s)}$ is the restriction of the execution context to dep(s). The function $input:S\times Z_{i}⇀D$ is partial: input(s,z) is defined if and only if $\xi_{z}\left(p\right)$ is defined for every p∈dep(s), so that the restriction $\xi_{z}{|}_{dep(s)}$ is total; otherwise, $\kappa_{s}$ is not applied. By Lemma 2, this holds whenever *s* is enabled or running. Only the concrete payload structure of *D* and the bodies of $\kappa_{s}$ are application-specific. $d_{s,z}=input\left(s,z\right)$ denotes the concrete input passed to the selected service provider.

### 5.5. Request Compilation

Every executable step is transformed into an ordinary Arrowhead request by the compilation function $compile_{i}:S\times Z_{i}⟶Req_{i}$.

For s∈S and $z\in Z_{i}$, let $req_{s,z}=compile_{i}\left(s,z\right)$.

The compiled request contains the static requirements associated with the step, θ(s),ι(s),μ(s),q(s), together with any runtime information required by the Arrowhead request representation $Req_{i}$ introduced in Section 3.

Abstractly,

$$compile_{i}\left(s,z\right)=\mathrm{request}\left(\theta (s),\iota (s),\mu (s),q(s),\lambda (s,z)\right),$$

where $\lambda :S\times Z_{i}\to \Lambda_{i}$ collects session-dependent request parameters, orchestration preferences, and other runtime constraints belonging to the Arrowhead request model $Req_{i}$ recalled in Section 3.

Thus, $compile_{i}\left(s,z\right)\in Req_{i}$ for every well-formed step and workflow session.

The compilation function does not select a provider. It only translates a step-level service requirement into the request representation expected by the Arrowhead Orchestrator.

### 5.6. Service Orchestration

Let $\Gamma_{i}$ denote the orchestration-constraint space of the Arrowhead Orchestrator recalled in Section 3, and let $\gamma_{s,z}\in \Gamma_{i}$ denote the constraints applicable to step *s* in Session *z*.

At time t∈T, the orchestration result for step *s* is $O_{i}\left(c_{z}\left(s\right),compile_{i}\left(s,z\right),\gamma_{s,z},t\right)\in \Pi_{i}\cup \left\{⊥\right\}$.

If orchestration succeeds, the result is a concrete orchestration plan $\pi_{s,z}\in \Pi_{i}$.

If no feasible and authorized provider can be found, the result is ⊥.

The workflow choreographer does not modify or supplement this decision. Authorization, QoS filtering, provider selection, and inter-cloud routing remain governed entirely by the Arrowhead orchestration function $O_{i}$ recalled in Section 3.

### 5.7. Service Invocation

A successful orchestration plan can be used to invoke the selected service. Invocation is represented by the abstract function $consume_{i}:\Pi_{i}\times D⟶D\cup \left\{\mathrm{fail}\right\}$.

For a plan $\pi_{s,z}\in \Pi_{i}$ and step input $d_{s,z}\in D$,

$$consume_{i}\left(\pi_{s,z},d_{s,z}\right)=\left\{\begin{array}{cc} d_{s,z}^{\prime }, & \mathrm{if}\;\mathrm{service}\;\mathrm{execution}\;\mathrm{succeeds},\\ \mathrm{fail}, & \mathrm{if}\;\mathrm{service}\;\mathrm{execution}\;\mathrm{fails}, \end{array}\right.$$

where $d_{s,z}^{\prime }\in D$ is the result returned by the service provider.

A call does not have to return at once. For example, ReserveTool of Section 8 waits until the tool has been released by its holder. We therefore read $consume_{i}\left(\pi ,d\right)=r$ as “the provider has answered the call with r∈D∪{fail}”. While a call is pending, no answer exists. The step keeps the marking running, and neither S-Done nor S-FailInvoke is applicable to it, because their premises require the answer. Other steps, also of other sessions, can still make transitions in the meantime. When the answer arrives, exactly one of the two rules applies and records it in one transition. A pending call is thus not a separate state component or rule: it is a step that is running and whose answer has not been given yet. The input of the call does not change while it is pending, because the results in dep(s) are completed and are not changed by any rule (Lemma 1).

The abstraction hides protocol-specific details such as HTTP, CoAP, MQTT, OPC UA, or other communication mechanisms supported by the selected service interface.

### 5.8. Fundamental Execution Primitive

Combining compilation, orchestration, and invocation, the execution of one step is characterized by

$$s\overset{compile_{i}\left(\;\cdot \;,z\right)}{\to }req_{s,z}\overset{O_{i}\left(c_{z}\left(s\right),\;\cdot \;,\gamma_{s,z},t\right)}{\to }\pi_{s,z}\overset{consume_{i}\left(\;\cdot \;,d_{s,z}\right)}{\to }d_{s,z}^{\prime }.$$

If either orchestration or invocation fails, the successful chain above is interrupted by $O_{i}\left(c_{z}\left(s\right),compile_{i}\left(s,z\right),\gamma_{s,z},t\right)=⊥$ or $consume_{i}\left(\pi_{s,z},d_{s,z}\right)=\mathrm{fail}$.

It is important to state that formalizing recovery behavior is outside of the scope of this model. A failed orchestration or invocation is captured by the failure rules S-FailBind and S-FailInvoke of Section 5.9, which only record the failure in the session status; retry and re-orchestration are not modeled.

Furthermore, unless stated otherwise, only the state components affected by the current execution step are displayed. All other components are carried over unchanged to the next state.

Altogether, this composition is the fundamental execution primitive of Arrowhead workflow choreography. All workflow behavior is obtained by applying this primitive to steps in an order permitted by the workflow recipe.

### 5.9. Transition Rules

The execution primitive is lifted to workflow sessions by a labeled transition relation $z\overset{\ell }{\to }z^{\prime }$ on $Z_{i}$. It is a *marking-migration system*: the step marking *M* and the action marking $M_{A}$ are the tags carried by steps and actions, and every rule migrates exactly one tag, or the session status. Parallel execution of independent steps is modeled by interleaving.

Conventions.

z[x↦v] denotes the state *z* in which component *x* is replaced by *v* and all other components are unchanged. Variables that occur only in the premises of a rule are existentially quantified. All rules apply only if status=running, and a transition occurs at a time t∈T, which is the time argument of $O_{i}$. For a step *s* and state *z*, the set of states after the assignment of a workflow executor is

$$asg\left(z,s\right)=\left\{\begin{array}{cc} \{z\}, & \eta (s)\;\mathrm{undefined},\\ \{\;z\left[\varepsilon \mapsto \varepsilon \left[s\mapsto w\right]\right]|w\in WE_{i},\;w⊧\eta \left(s\right)\;\}, & \eta (s)\;\mathrm{defined}. \end{array}\right.$$

The rules assume that qnty(s)=1 for every step, i.e., each step is executed once; repeated execution is not modeled.

Rules.

$$\frac{\begin{array}{c} M_{A}\left(\alpha \right)=\mathrm{inactive}\;\left(\alpha^{\prime },\alpha \right)\in next_{A}\;M_{A}\left(\alpha^{\prime }\right)=\mathrm{completed} \end{array}}{z\overset{\mathrm{start}(\alpha )}{\to }z\left[M_{A}\left(\alpha \right)\mapsto \mathrm{active}\right]}\;A-\mathrm{START}$$

$$\frac{\begin{array}{c} M_{A}\left(\alpha \left(s\right)\right)=\mathrm{active}\;M\left(s\right)=\mathrm{inactive}\\ \forall p\in pre(s):\;M(p)=\mathrm{completed} \end{array}}{z\overset{\mathrm{enable}(s)}{\to }z\left[M\left(s\right)\mapsto \mathrm{enabled}\right]}\;S-\mathrm{ENABLE}$$

$$\frac{\begin{array}{c} M\left(s\right)=\mathrm{enabled}\;z^{\prime }\in asg\left(z,s\right)\\ \pi =O_{i}\left(c_{z^{\prime }}\left(s\right),\;compile_{i}\left(s,z^{\prime }\right),\;\gamma_{s,z^{\prime }},\;t\right)\neq ⊥ \end{array}}{z\overset{\mathrm{bind}(s,\pi )}{\to }z^{\prime }\left[M\left(s\right)\mapsto \mathrm{running},\;\beta \left(s\right)\mapsto \pi \right]}\;S-\mathrm{BIND}$$

$$\frac{\begin{array}{c} M(s)=\mathrm{running}\;\beta (s)=\pi \\ consume_{i}\left(\pi ,\;input(s,z)\right)=d^{\prime }\neq \mathrm{fail} \end{array}}{z\overset{\mathrm{done}(s)}{\to }z\left[M\left(s\right)\mapsto \mathrm{completed},\;\xi \left(s\right)\mapsto d^{\prime }\right]}\;S-\mathrm{DONE}$$

$$\frac{M_{A}\left(\alpha \right)=\mathrm{active}\;\forall s\in S_{\alpha }:\;M\left(s\right)=\mathrm{completed}}{z\overset{\mathrm{finish}(\alpha )}{\to }z\left[M_{A}\left(\alpha \right)\mapsto \mathrm{completed}\right]}\;A-\mathrm{FINISH}$$

$$\frac{\forall \alpha \in A:\;M_{A}\left(\alpha \right)=\mathrm{completed}}{z\overset{\mathrm{close}}{\to }z\left[status\mapsto \mathrm{completed}\right]}\;Z-\mathrm{CLOSE}$$

$$\frac{\begin{array}{c} M(s)=\mathrm{enabled}\\ \forall z^{\prime }\in asg\left(z,s\right):\;O_{i}\left(c_{z^{\prime }}\left(s\right),compile_{i}\left(s,z^{\prime }\right),\gamma_{s,z^{\prime }},t\right)=⊥ \end{array}}{z\overset{\mathrm{fail}(s)}{\to }z\left[status\mapsto \mathrm{failed}\right]}\;S-\mathrm{FAILBIND}$$

$$\frac{M\left(s\right)=\mathrm{running}\;\beta \left(s\right)=\pi \;consume_{i}\left(\pi ,\;input(s,z)\right)=\mathrm{fail}}{z\overset{\mathrm{fail}(s)}{\to }z\left[status\mapsto \mathrm{failed}\right]}\;S-\mathrm{FAILINVOKE}$$

Note that asg(z,s)=⌀ (no workflow executor satisfies η(s)) makes the premise of S-FailBind vacuously true. The failure rules are absorbing: after them, no rule applies, and recovery is outside the scope of the model. Rule S-Bind realizes the first two stages of the execution primitive ($compile_{i}$ and $O_{i}$), and S-Done realizes $consume_{i}$ together with the state update.

A step whose call is still pending stays running and enables neither of the two rules (Section 5.7); this is how a waiting call such as ReserveTool is represented.

Properties.

A *reachable* state is a state reachable from the initial state $z_{0}$ by a run (Section 5.10).

**Lemma** **1** (Monotonicity and enabling safety)**.** *In every reachable state: (i) markings never decrease along a run, where inactive<enabled<running<completed for steps and inactive<active<completed for actions; (ii) M(s)≠inactive implies that M(p)=completed for all p∈pre(s), and $M_{A}\left(\alpha \left(s\right)\right)\neq \mathrm{inactive}$; (iii) M(s)=completed implies that ξ(s) is defined; (iv) M(s)≠inactive implies that M(p)=completed for every p≺s; (v) the executor assignment ε is valid in the sense of Section 5.2.*

**Proof.**  (i) Every rule replaces a marking by a larger one. (ii) A step leaves inactive only by S-Enable, whose premises give the claim; by (i), completed steps stay completed and active actions never return to inactive. (iii) S-Done is the only rule that completes a step, and it defines ξ(s); no rule removes a value from ξ. (iv) Let $p\in pre^{+}\left(s\right)$. Then, there is a chain $s=s_{0},s_{1},\ldots ,s_{n}=p$ with $s_{k+1}\in pre\left(s_{k}\right)$. By (ii), $M(s_{0})\neq \mathrm{inactive}$ gives $M(s_{1})=\mathrm{completed}$; hence, $M(s_{1})\neq \mathrm{inactive}$, and inductively M(p)=completed. Now let $\alpha \left(p\right)=\alpha_{i}$ and $\alpha \left(s\right)=\alpha_{j}$ with i<j on the path $\alpha_{0}\to \cdots \to \alpha_{n}$. By (ii), $M_{A}\left(\alpha_{j}\right)\neq \mathrm{inactive}$. An action other than $\alpha_{0}$ leaves inactive only by A-Start, whose premise requires its predecessor to be completed. Hence, $M_{A}\left(\alpha_{j-1}\right)=\mathrm{completed}$ by (i), and inductively $M_{A}\left(\alpha_{i}\right)=\mathrm{completed}$. An action is completed only by A-Finish, which requires all its steps to be completed. Hence, M(p)=completed. (v) The initial assignment is empty, hence valid. Among the rules, only S-Bind changes ε, and it does so through $z^{\prime }\in asg\left(z,s\right)$: if η(s) is undefined, then $z^{\prime }=z$ and (V1)–(V3) are unchanged for *s*; if η(s) is defined, then $z^{\prime }$ assigns to *s* an executor *w* with w⊧η(s), which gives (V1) and (V2). A step becomes running only by S-Bind, in the same transition in which this assignment is made, and ε is not changed by any other rule, which gives (V3). □

**Lemma** **2** (Input well-definedness)**.** *Let W be well-formed. In every reachable state with M(s)≠inactive, the value ξ(p) is defined for every p∈dep(s). Hence, input(s,z) is defined whenever s is enabled or running.*

**Proof.**  Let p∈dep(s). By (W4), p≺s. Since M(s)≠inactive, Lemma 1(iv) gives M(p)=completed, and Lemma 1(iii) shows that ξ(p) is defined. □

**Theorem** **1** (Bounded runs)**.** *Every run of a workflow session of a well-formed recipe has at most 3|S|+2|A| transitions. A failure-free run that ends with* Z-Close *has exactly 3|S|+2|A| transitions.*

**Proof.**  Assign rank 0,1,2,3 to the step markings inactive,enabled,running,completed and rank 0,1,2 to the action markings inactive,active,completed, and let Φ(z) be the sum of all ranks. S-Enable, S-Bind, S-Done, A-Start and A-Finish increase Φ by exactly 1, no rule decreases it, and Φ≤3|S|+2|A|. Since $\alpha_{0}$ is active in $z_{0}$, $\Phi (z_{0})\geq 1$, so at most 3|S|+2|A|−1 transitions increase Φ. The rules Z-Close, S-FailBind and S-FailInvoke change only status, and after any of them, no rule applies, so together they contribute at most one transition, which gives the bound. In a failure-free run that ends with Z-Close, all actions are completed, and hence all steps are completed, so Φ reaches 3|S|+2|A| and the run has exactly (3|S|+2|A|−1)+1 transitions. □

**Theorem** **2** (Progress)**.** *Let W be well-formed and z a reachable state with status=running. Then, at least one rule is applicable in z. Consequently, every maximal run ends in a state with status=completed or status=failed.*

**Proof.**  If all actions are completed, Z-Close applies. Otherwise, let $\alpha_{k}$ be the first non-completed action on the path $\alpha_{0}\to \cdots \to \alpha_{n}$ (well-formedness (W1)). If $M_{A}\left(\alpha_{k}\right)=\mathrm{inactive}$, then k≥1, because $\alpha_{0}$ is active in $z_{0}$ and markings do not decrease, and $\alpha_{k-1}$ is completed, so A-Start applies. If $M_{A}\left(\alpha_{k}\right)=\mathrm{active}$ and all its steps are completed, A-Finish applies. Otherwise, the set of non-completed steps of $\alpha_{k}$ is finite and, because $G_{\alpha_{k}}$ is acyclic, contains a step *s* all of whose predecessors are completed. If M(s)=inactive, S-Enable applies. If M(s)=enabled, then S-Bind or S-FailBind applies. If M(s)=running, then S-Done or S-FailInvoke applies, where input(s,z) is defined by Lemma 2. □

Theorems 1 and 2 state termination and deadlock-freedom of workflow sessions as properties of the transition system. They hold for the model without recovery and with qnty(s)=1. Repeated execution of a step and re-orchestration after a failed or revoked binding are not covered by the rules. Theorem 2 also presupposes that every service invocation eventually returns a result or fail, i.e., that $consume_{i}$ is total. Section 8 shows a case in which this depends on the provider.

### 5.10. Execution Trace Notation

The execution model introduced above defines the runtime state of a workflow session, including step markings, action markings, service bindings, executor assignments, execution contexts, and the overall session status. A *run* of a workflow session is a sequence $z_{0}\overset{\ell_{1}}{\to }z_{1}\overset{\ell_{2}}{\to }\cdots \overset{\ell_{n}}{\to }z_{n}$, where $z_{0}$ is the initial state and every transition is an instance of a rule of Section 5.9. The label $\ell_{k}$ names the rule instance: start(α), enable(s), bind(s,π), done(s), finish(α), close or fail(s). A run is *maximal* if no rule applies in $z_{n}$. In the worked examples below, every arrow is annotated by its rule, and the failure-free maximal run is shown. For a step *s*, the marking M(s) records its runtime status, while β(s) stores the current service binding, if any. Thus, β(s)=⊥ denotes that no binding has yet been assigned to *s*, whereas β(s)=π denotes that step *s* is bound to the service instance described by π. The examples focus on the normal execution path: recovery after failure, re-orchestration, and repeated execution (qnty>1) are outside the scope of this presentation.

Table 4 summarizes the symbols introduced for the workflow session and the workflow execution model in Section 5.

### 5.11. What the Model Guarantees in Practice

The formal model is not only a notation. It makes four statements that a choreographer implementation, a recipe author or an operator can check, and that could not be stated precisely from the informal description of the workflow choreography.

**(a) Recipes can be validated before any session runs.** Conditions (W1)–(W4) are structural, so they can be checked in time polynomial in the size of the recipe. A recipe with an action that is not reachable from $\alpha_{0}$, a cyclic step graph, or a data dependency on a step that does not precede the consumer is rejected before execution, instead of blocking a session at runtime.

**(b) A step never runs on missing data.** By Lemma 1(iv) and Lemma 2, every step that is enabled or running has all the steps that it depends on completed, and their results stored in ξ. For a measurement workflow, this means that an evaluation or actuation step never starts before the steps that produce the measurement it consumes have finished. This holds across actions too.

**(c) Every session terminates or fails explicitly, and the number of its transitions is known.** By Theorems 1 and 2, a session never deadlocks, provided that every service invocation returns a result or a failure, and a failure-free session of a recipe with |S| steps and |A| actions has exactly 3|S|+2|A| transitions. This bounds the number of transitions, not the duration of a session: a service call can stay pending without adding any transition. The count can therefore be used as a progress indicator and as a consistency check of the logs, but not as a timeout. A timeout needs a separate timing rule, for example, a maximum waiting time for a running step after which its call is treated as fail. Such a rule is not part of the model.

**(d) Shared resources can be protected by the recipe structure.** Proposition 1 shows that if the tool manager grants a resource to one session at a time, the recipe’s action order makes all uses of the resource by different sessions disjoint in time.

Table 5 relates each statement to its formal basis and to the place where the paper demonstrates it. Statements (b)–(d) hold for the model without recovery and with qnty(s)=1 (Section 5.9). The model does not give a quantitative prediction of latency or throughput, because the transition system abstracts from step durations.

### 5.12. Executable Demonstrators and Mapping to the Choreographer

Both worked examples are available as interactive simulations and as runnable kits [42]. In a simulation, the user acts as the scheduler: every button is one rule instance of Section 5.9 that is applicable in the current session state, so each session is a labeled run of the transition system. On the executed runs, the simulations check Lemma 2, the bound of Theorem 1, progress and, for the tool delivery example, Proposition 1. They also inject disturbances that trigger the failure rules: a provider that is not registered or an executor that is offline (S-FailBind, in the latter case with asg(z,s)=⌀), and a failed invocation (S-FailInvoke). The simulation of the temperature-control example additionally lets the room cool faster when a window is open, and the simulation of the tool delivery example lets the torque of one bolt drift.

The kits register the recipes at the workflow choreographer of Eclipse Arrowhead release 4.6.2 and execute them with application systems that simulate the plant. Both examples were executed on this choreographer, and the logs of these executions were compared with the runs of the transition system.

We compared 20 sessions of the temperature-control example (20 control periods, 10 of them with the broken switch) and 50 sessions of the tool delivery example (20 with no special options, 10 with concurrent line sessions, 10 with the AGV route blocked and 10 with the lease option). For each session, we checked that (i) the order of the step executions is a sequence of rule instances S-Enable, S-Bind, and S-Done that the model permits, (ii) the results stored in ξ are the results returned by the providers, and (iii) in the failure cases, the session ends with the rule S-FailInvoke or S-FailBind that the model predicts, and no later step is started. In all 50 sessions, the order of the steps, the stored results and the failures agreed with the model.

Table 6 states how the elements of the model are realized in the kits.

In the temperature-control example, η is undefined for every step, so in the model, the consumer is $c_{z}\left(s\right)=CH_{i}$. In the kits, the choreographer of release 4.6.2 starts every step through a registered executor, so the steps of this example also run through executors ($ex_{\mathrm{read}}$, $ex_{\mathrm{evaluate}}$, $ex_{\mathrm{switch}}$). These executors do not offer a capability e∈E that the model needs for selection; they are the technical form in which the choreographer executes the step itself. In the tool delivery example, η is defined only for the two AGV steps, and the other steps are realized in the same way. Consequently, when the kit is compared with the model, the consumer identity of the model ($CH_{i}$ or the assigned executor) is represented by the identity of the executor that runs the step. Authorization follows this: in the kits, an authorization rule is created per executor for its own service, for the session-context service and for its declared dependencies, and only the executor agvexecutor may consume TransportTool, which is (A2). The authorization of $c_{z}\left(s\right)$ in the model and the rules in the kit therefore agree up to this renaming.

## 6. Worked Example—A Generic Illustration

This section illustrates the proposed mathematical description on a small workflow recipe. The example is intentionally simple: its purpose is not to introduce additional semantics, but to show how the previously defined recipe and execution model are instantiated in a concrete workflow execution.

We consider a generic industrial monitoring-and-control workflow. A sensor value is first acquired and then evaluated, and finally an actuator command is issued if intervention is required.

The notation conventions are the following, for both worked examples.

In both worked examples, the three steps are denoted $s_{\mathrm{read}}$, $s_{\mathrm{eval}}$ and $s_{\mathrm{act}}$ (acquire, evaluate, actuate); the second example gives them temperature-specific service definitions $\theta_{\mathrm{read}},\theta_{\mathrm{eval}},\theta_{\mathrm{act}}$. For the *k*-th step (k=1,2,3) executed in state *z*, the compiled request, the selected binding, the input and the result are written as $req_{k}=req_{s,z}$, $\pi_{k}=\pi_{s,z}$, $d_{k}=d_{s,z}$ and $d_{k}^{\prime }=d_{s,z}^{\prime }$, respectively, as in Section 5.

The *j*-th state of a run is $z_{j}$, and $M_{j},\;M_{A,j},\;\beta_{j},\;\xi_{j},\;status_{j}$ denote its components. The consumer is $c_{z}\left(s\right)=CH_{i}$ throughout, since no step requires a workflow executor.

### 6.1. Example Scenario

The workflow consists of one action that contains three steps: $s_{\mathrm{read}},\;\;s_{\mathrm{eval}},\;\;s_{\mathrm{act}}$.

The intended meanings of these steps are:

- $s_{\mathrm{read}}$: read a measurement from a sensor service;
- $s_{\mathrm{eval}}$: evaluate the measurement;
- $s_{\mathrm{act}}$: invoke an actuator service if required.

The steps must be executed in the order $s_{\mathrm{read}}\;⟶\;s_{\mathrm{eval}}\;⟶\;s_{\mathrm{act}}$.

Thus, the example captures a typical sensing–decision–actuation pattern, while remaining independent of any concrete industrial domain.

### 6.2. Workflow Recipe Instance

Let the workflow recipe contain one action $A=\{\alpha_{\mathrm{ctrl}}\}$, where $\alpha_{\mathrm{ctrl}}$ denotes the complete monitoring-and-control procedure. Since this is the only action, it is also the initial action: $\alpha_{0}=\alpha_{\mathrm{ctrl}}$.

The action-transition relation is empty, $next_{A}=⌀$, because there are no further actions after $\alpha_{\mathrm{ctrl}}$. Therefore, the plan is $P=(\mathrm{MonitoringControl},A,\alpha_{0},next_{A})$.

The step set of action $\alpha_{\mathrm{ctrl}}$ is $S_{\alpha_{\mathrm{ctrl}}}=\left\{s_{\mathrm{read}},s_{\mathrm{eval}},s_{\mathrm{act}}\right\}$.

The internal step-dependency relation of the action is

$$next_{\alpha_{\mathrm{ctrl}}}=\left\{\left(s_{\mathrm{read}},s_{\mathrm{eval}}\right),\left(s_{\mathrm{eval}},s_{\mathrm{act}}\right)\right\}.$$

Hence, $F_{\alpha_{\mathrm{ctrl}}}=\left\{s_{\mathrm{read}}\right\}$, because $s_{\mathrm{read}}$ is the only step without predecessors. The action itself is therefore

$$\alpha_{\mathrm{ctrl}}=\left(S_{\alpha_{\mathrm{ctrl}}},F_{\alpha_{\mathrm{ctrl}}},next_{\alpha_{\mathrm{ctrl}}}\right).$$

The complete step universe of the recipe is $S=S_{\alpha_{\mathrm{ctrl}}}$.

The predecessor function is given by $pre(s_{\mathrm{read}})=⌀$, $pre\left(s_{\mathrm{eval}}\right)=\left\{s_{\mathrm{read}}\right\}$, and $pre\left(s_{\mathrm{act}}\right)=\left\{s_{\mathrm{eval}}\right\}$.

Data dependencies.

Let the session parameters be $\rho =(\rho_{\mathrm{req}},\rho_{\mathrm{thr}})$, a measurement request and a decision threshold. The steps consume the result of their direct predecessor,

$$dep\left(s_{\mathrm{read}}\right)=⌀,\;dep\left(s_{\mathrm{eval}}\right)=\left\{s_{\mathrm{read}}\right\},\;dep\left(s_{\mathrm{act}}\right)=\left\{s_{\mathrm{eval}}\right\},$$

which satisfies $dep\left(s\right)\subseteq pre^{+}\left(s\right)$. With $r\in D^{dep(s)}$, the input-composition functions are

$$\kappa_{s_{\mathrm{read}}}\left(\rho \right)=\rho_{\mathrm{req}},\;\kappa_{s_{\mathrm{eval}}}\left(\rho ,r\right)=\left(r\left(s_{\mathrm{read}}\right),\;\rho_{\mathrm{thr}}\right),\;\kappa_{s_{\mathrm{act}}}\left(\rho ,r\right)=r\left(s_{\mathrm{eval}}\right).$$

The service requirements of the three steps are $\theta (s_{\mathrm{read}})=\mathrm{ReadMeasurement}$, $\theta (s_{\mathrm{eval}})=\mathrm{EvaluateMeasurement}$, and $\theta (s_{\mathrm{act}})=\mathrm{ActuateDevice}$.

For simplicity, assume that all three steps use the same required interface $\iota_{0}$, unconstrained metadata $\mu_{0}$, and unconstrained QoS requirements $q_{0}$: $\iota \left(s\right)=\iota_{0},\;\;\mu \left(s\right)=\mu_{0},\;\;q\left(s\right)=q_{0},\;\;s\in S$.

No dedicated workflow executor capability is required in this example. Hence, η(s) is undefined for every s∈S.

Each step is executed once: qnty(s)=1,s∈S.

The complete workflow recipe is therefore W=(P,A,S,θ,ι,μ,q,pre,η,qnty,dep,κ).

### 6.3. Initial Workflow Session State

Let $z_{0}\in Z_{i}$ denote the initial workflow session state in Local Cloud $LC_{i}$. We write $M_{k}$, $\beta_{k}$, and $status_{k}$ for the step-marking component, the binding component, and the session-status component of state $z_{k}$, respectively.

Initially, no step has been executed and no service binding has been assigned: $M_{0}\left(s\right)=\mathrm{inactive},\;\;\beta_{0}\left(s\right)=⊥,\;\;s\in S$.

The workflow session is running: $status_{0}=\mathrm{running}$. The only action is active in the initial state, $M_{A,0}\left(\alpha_{\mathrm{ctrl}}\right)=\mathrm{active}$, and no result is stored yet; $\xi_{0}\left(s\right)$ is undefined for s∈S.

Since $pre(s_{\mathrm{read}})=⌀$, the first step that may become enabled is $s_{\mathrm{read}}$.

**Figure 4:**
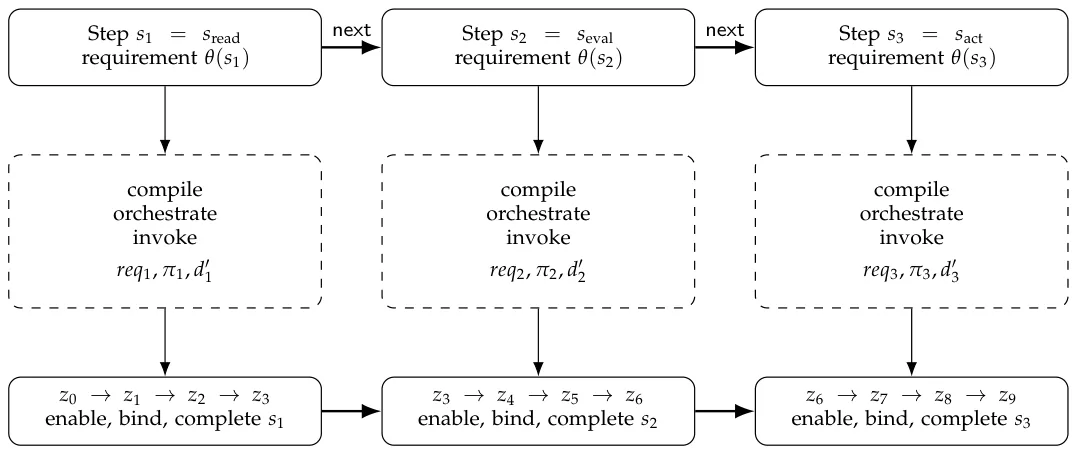
The generic workflow execution example illustrated as a compact state-indexed execution trace. The recipe contains three ordered steps. Each step is enabled, compiled into an Arrowhead orchestration request, resolved into a binding by the Orchestrator, invoked, and then completed before the next step becomes enabled. NOTE: The arrows labeled “next” represent the step-dependency relation ${\mathrm{next}}_{\alpha_{\mathrm{ctrl}}}$ inside the action $\alpha_{\mathrm{ctrl}}$.

Figure 4 illustrates a generic recipe execution in a workflow with three steps, each requiring orchestration.

### 6.4. Execution of the First Step

The execution starts by enabling the first step:

$$M_{1}\left(s_{\mathrm{read}}\right)=\mathrm{enabled}.$$

Transition $z_{0}\overset{\mathrm{enable}(s_{\mathrm{read}})}{\to }z_{1}$ (rule S-Enable).

The step is then transformed into an ordinary Arrowhead orchestration request: $req_{1}=compile_{i}\left(s_{\mathrm{read}},z_{1}\right)$.

Since no dedicated workflow executor is required, the consumer responsible for the step is the workflow choreographer itself: $c_{z_{1}}\left(s_{\mathrm{read}}\right)=CH_{i}$.

At time $t_{1}$, the Orchestrator returns a feasible service binding:

$$O_{i}\left(c_{z_{1}}\left(s_{\mathrm{read}}\right),req_{1},\gamma_{s_{\mathrm{read}},z_{1}},t_{1}\right)=\pi_{1},\;\pi_{1}\in \Pi_{i}.$$

The selected binding is stored in the workflow session state: $\beta_{2}\left(s_{\mathrm{read}}\right)=\pi_{1}$. This is the transition $z_{1}\overset{\mathrm{bind}(s_{\mathrm{read}},\pi_{1})}{\to }z_{2}$ (rule S-Bind); it also sets $M_{2}\left(s_{\mathrm{read}}\right)=\mathrm{running}$.

The input data for the step are $d_{1}=input\left(s_{\mathrm{read}},z_{2}\right)=\kappa_{s_{\mathrm{read}}}\left(\rho \right)=\rho_{\mathrm{req}}$.

The selected service is then invoked: $consume_{i}\left(\pi_{1},d_{1}\right)=d_{1}^{\prime },\;\;d_{1}^{\prime }\in D$.

The result $d_{1}^{\prime }$ represents the measurement returned by the selected sensor service. After successful invocation, the step is completed: $M_{3}\left(s_{\mathrm{read}}\right)=\mathrm{completed}$. This is the transition $z_{2}\overset{\mathrm{done}(s_{\mathrm{read}})}{\to }z_{3}$ (rule S-Done); it also stores the result, $\xi_{3}\left(s_{\mathrm{read}}\right)=d_{1}^{\prime }$.

### 6.5. Execution of the Second Step

The second step may execute after its predecessor has completed. Since $pre\left(s_{\mathrm{eval}}\right)=\left\{s_{\mathrm{read}}\right\}$ and $M_{3}\left(s_{\mathrm{read}}\right)=\mathrm{completed}$, the step $s_{\mathrm{eval}}$ becomes enabled: $M_{4}\left(s_{\mathrm{eval}}\right)=\mathrm{enabled}$. Transition $z_{3}\overset{\mathrm{enable}(s_{\mathrm{eval}})}{\to }z_{4}$ (rule S-Enable).

The workflow choreographer compiles $s_{\mathrm{eval}}$ into an orchestration request: $req_{2}=compile_{i}\left(s_{\mathrm{eval}},z_{4}\right)$.

The Orchestrator selects a feasible binding:

$$O_{i}\left(c_{z_{4}}\left(s_{\mathrm{eval}}\right),req_{2},\gamma_{s_{\mathrm{eval}},z_{4}},t_{2}\right)=\pi_{2},\;\pi_{2}\in \Pi_{i}.$$

The binding is stored as $\beta_{5}\left(s_{\mathrm{eval}}\right)=\pi_{2}$. This is the transition $z_{4}\overset{\mathrm{bind}(s_{\mathrm{eval}},\pi_{2})}{\to }z_{5}$ (rule S-Bind); it also sets $M_{5}\left(s_{\mathrm{eval}}\right)=\mathrm{running}$.

The input of $s_{\mathrm{eval}}$ is composed from the result produced by $s_{\mathrm{read}}$ and from the threshold parameter:

$$d_{2}=input\left(s_{\mathrm{eval}},z_{5}\right)=\kappa_{s_{\mathrm{eval}}}\left(\rho ,\xi_{5}{|}_{dep(s_{\mathrm{eval}})}\right)=\left(\xi_{5}\left(s_{\mathrm{read}}\right),\rho_{\mathrm{thr}}\right)=\left(d_{1}^{\prime },\rho_{\mathrm{thr}}\right).$$

The selected evaluation service is invoked: $consume_{i}\left(\pi_{2},d_{2}\right)=d_{2}^{\prime },\;\;d_{2}^{\prime }\in D$.

The result $d_{2}^{\prime }$ represents the evaluation outcome. The step then completes: $M_{6}\left(s_{\mathrm{eval}}\right)=\mathrm{completed}$. This is the transition $z_{5}\overset{\mathrm{done}(s_{\mathrm{eval}})}{\to }z_{6}$ (rule S-Done); it also stores the result, $\xi_{6}\left(s_{\mathrm{eval}}\right)=d_{2}^{\prime }$.

### 6.6. Execution of the Third Step

The third step may execute after $s_{\mathrm{eval}}$ has completed. Since $pre\left(s_{\mathrm{act}}\right)=\left\{s_{\mathrm{eval}}\right\}$ and $M_{6}\left(s_{\mathrm{eval}}\right)=\mathrm{completed}$, the step $s_{\mathrm{act}}$ becomes enabled: $M_{7}\left(s_{\mathrm{act}}\right)=\mathrm{enabled}$. Transition $z_{6}\overset{\mathrm{enable}(s_{\mathrm{act}})}{\to }z_{7}$ (rule S-Enable).

The step is compiled into an Arrowhead request: $req_{3}=compile_{i}\left(s_{\mathrm{act}},z_{7}\right)$.

At time $t_{3}$, the Orchestrator returns a feasible actuator-service binding:

$$O_{i}\left(c_{z_{7}}\left(s_{\mathrm{act}}\right),req_{3},\gamma_{s_{\mathrm{act}},z_{7}},t_{3}\right)=\pi_{3},\;\pi_{3}\in \Pi_{i}.$$

The binding is stored in the workflow session state: $\beta_{8}\left(s_{\mathrm{act}}\right)=\pi_{3}$. This is the transition $z_{7}\overset{\mathrm{bind}(s_{\mathrm{act}},\pi_{3})}{\to }z_{8}$ (rule S-Bind); it also sets $M_{8}\left(s_{\mathrm{act}}\right)=\mathrm{running}$.

The input of the actuator step is composed from the evaluation result:

$$d_{3}=input\left(s_{\mathrm{act}},z_{8}\right)=\kappa_{s_{\mathrm{act}}}\left(\rho ,\xi_{8}{|}_{dep(s_{\mathrm{act}})}\right)=\xi_{8}\left(s_{\mathrm{eval}}\right)=d_{2}^{\prime }.$$

The selected actuator service is invoked: $consume_{i}\left(\pi_{3},d_{3}\right)=d_{3}^{\prime },\;\;d_{3}^{\prime }\in D$.

After successful invocation, the actuator step completes: $M_{9}\left(s_{\mathrm{act}}\right)=\mathrm{completed}$. This is the transition $z_{8}\overset{\mathrm{done}(s_{\mathrm{act}})}{\to }z_{9}$ (rule S-Done); it also stores the result, $\xi_{9}\left(s_{\mathrm{act}}\right)=d_{3}^{\prime }$.

### 6.7. Final Workflow Session State

After $z_{9}$, all steps have completed, $M_{9}\left(s\right)=\mathrm{completed}$ for s∈S, but the session is still running, $status_{9}=\mathrm{running}$. The action and the session are then closed by the rules A-Finish and Z-Close: $M_{A,10}\left(\alpha_{\mathrm{ctrl}}\right)=\mathrm{completed}$ and $status_{11}=\mathrm{completed}$. The complete run, in which every arrow is an instance of the indicated rule of Section 5.9, is

$$\begin{array}{cc} z_{0} & \overset{\mathrm{enable}(s_{\mathrm{read}})}{\to }z_{1}\overset{\mathrm{bind}(s_{\mathrm{read}},\pi_{1})}{\to }z_{2}\overset{\mathrm{done}(s_{\mathrm{read}})}{\to }z_{3}\\ & \overset{\mathrm{enable}(s_{\mathrm{eval}})}{\to }z_{4}\overset{\mathrm{bind}(s_{\mathrm{eval}},\pi_{2})}{\to }z_{5}\overset{\mathrm{done}(s_{\mathrm{eval}})}{\to }z_{6}\\ & \overset{\mathrm{enable}(s_{\mathrm{act}})}{\to }z_{7}\overset{\mathrm{bind}(s_{\mathrm{act}},\pi_{3})}{\to }z_{8}\overset{\mathrm{done}(s_{\mathrm{act}})}{\to }z_{9}\\ & \overset{\mathrm{finish}(\alpha_{\mathrm{ctrl}})}{\to }z_{10}\overset{\mathrm{close}}{\to }z_{11}. \end{array}$$

Table 7 lists this run state by state.

**Table 7:** Illustrative execution trace of the example workflow recipe.

State	Operation	Main Effect
$z_{0}$	Initial state	All steps are inactive and unbound.
$z_{1}$	Enable $s_{\mathrm{read}}$	$M_{1}\left(s_{\mathrm{read}}\right)=\mathrm{enabled}$.
$z_{2}$	Orchestrate $s_{\mathrm{read}}$	$\beta_{2}\left(s_{\mathrm{read}}\right)=\pi_{1}$.
$z_{3}$	Invoke and complete $s_{\mathrm{read}}$	$M_{3}\left(s_{\mathrm{read}}\right)=\mathrm{completed}$.
$z_{4}$	Enable $s_{\mathrm{eval}}$	$M_{4}\left(s_{\mathrm{eval}}\right)=\mathrm{enabled}$.
$z_{5}$	Orchestrate $s_{\mathrm{eval}}$	$\beta_{5}\left(s_{\mathrm{eval}}\right)=\pi_{2}$.
$z_{6}$	Invoke and complete $s_{\mathrm{eval}}$	$M_{6}\left(s_{\mathrm{eval}}\right)=\mathrm{completed}$.
$z_{7}$	Enable $s_{\mathrm{act}}$	$M_{7}\left(s_{\mathrm{act}}\right)=\mathrm{enabled}$.
$z_{8}$	Orchestrate $s_{\mathrm{act}}$	$\beta_{8}\left(s_{\mathrm{act}}\right)=\pi_{3}$.
$z_{9}$	Invoke and complete $s_{\mathrm{act}}$	$M_{9}\left(s_{\mathrm{act}}\right)=\mathrm{completed}$, all steps are completed.
$z_{10}$	Finish $\alpha_{\mathrm{ctrl}}$ (A-Finish)	$M_{A,10}\left(\alpha_{\mathrm{ctrl}}\right)=\mathrm{completed}$.
$z_{11}$	Close the Session (Z-Close)	$status_{11}=\mathrm{completed}$.

## 7. Worked Example—A Simple Demonstration

This section illustrates the mathematical model through a temperature-control workflow executed in one Arrowhead Local Cloud $LC_{i}$. An interactive simulation of this example, in which every transition is an instance of a rule of Section 5.9, is available at [42].

The running demonstration is available at: https://arrowhead-workflows.vercel.app/temperature-control (accessed on 2 November 2026).

The Local Cloud contains the following application systems:

(i) A temperature sensor;
(ii) A temperature controller;
(iii) A heating switch.

The workflow periodically reads the current temperature, evaluates it against a threshold of 23 °C, and switches the heating off whenever the measured temperature exceeds that threshold. We describe one execution period as one workflow session. The notation of the previous example is used: the steps are $s_{\mathrm{read}}$, $s_{\mathrm{eval}}$, $s_{\mathrm{act}}$ (the last one switches the heating), and $req_{k}$, $\pi_{k}$, $d_{k}$, $d_{k}^{\prime }$ refer to the *k*-th step (k=1,2,3).

**Figure 5:**
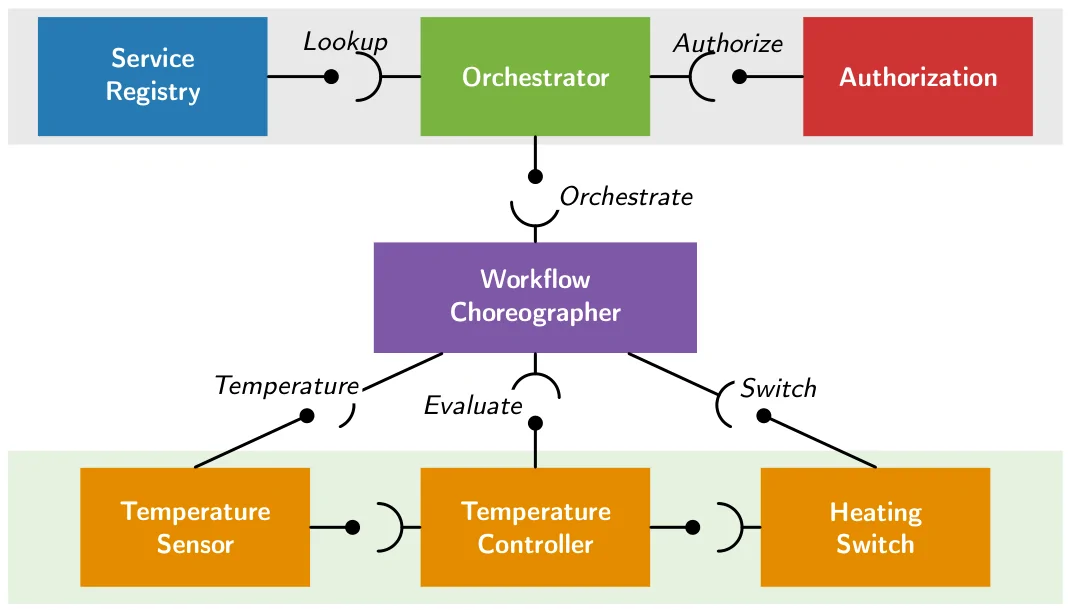
Architectural setup of core systems and application systems used in the temperature-control demonstration. Illustrative example: application systems can exchange services directly as well, but during the recipe execution, information exchange is done through the workflow choreographer instead.

Figure 5 shows the architectural setup of the core systems and application systems used in this example.

### 7.1. Application Systems and Services

Let $AS_{\mathrm{sensor}},\;\;AS_{\mathrm{controller}},\;\;AS_{\mathrm{switch}}$ denote the three application systems. They provide the service definitions $\theta_{\mathrm{read}}=\mathrm{ReadTemperature}$, $\theta_{\mathrm{eval}}=\mathrm{EvaluateTemperature}$, and $\theta_{\mathrm{act}}=\mathrm{SwitchHeating}$.

The associated logical operations are

- ReadTemperature()⟶temp;
- EvaluateTemperature(temp,23)⟶decision;
- SwitchHeating(decision)⟶switchState.

The application data domain contains at least D⊇R∪{true,false,ON,OFF}.

**Figure 6:**
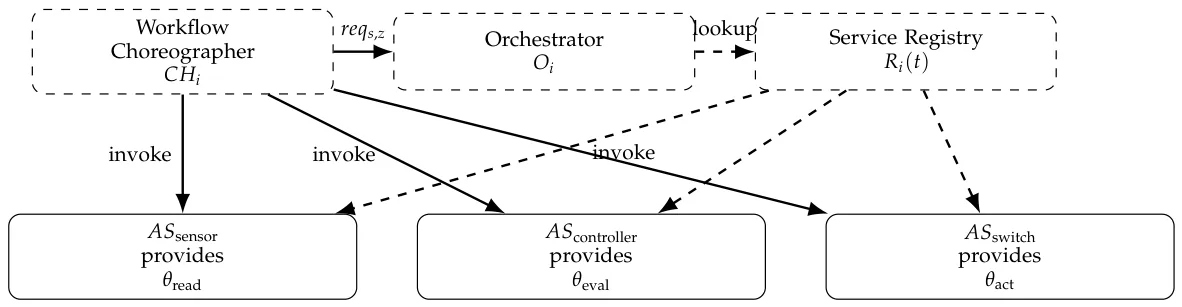
Application systems and services used in the temperature-control demonstration. The workflow choreographer compiles steps into Arrowhead orchestration requests, the Orchestrator resolves the requests using the Service Registry, and the selected application systems provide the required services. Note that completing authorization services is a pre-requisite; hence, the related core system is not shown.

Here, temp∈R is the measured temperature, decision is a Boolean value indicating whether the heating should be switched off, and switchState∈{ON,OFF} is the resulting state of the heating switch. Figure 6 shows how these application systems and their services are consumed by the workflow choreographer through the Orchestrator and Service Registry.

### 7.2. Workflow Recipe Instance

The workflow recipe contains one action $A=\{\alpha_{\mathrm{temp}}\}$, where $\alpha_{\mathrm{temp}}$ denotes the complete temperature-control procedure. Since the recipe contains only this action, the initial action is $\alpha_{0}=\alpha_{\mathrm{temp}}$, and the action-transition relation is empty: $next_{A}=⌀$.

Thus, the plan is $P=(\mathrm{TemperatureControl},A,\alpha_{0},next_{A})$.

The action $\alpha_{\mathrm{temp}}$ consists of three steps: $S_{\alpha_{\mathrm{temp}}}=\left\{s_{\mathrm{read}},s_{\mathrm{eval}},s_{\mathrm{act}}\right\}$.

The intended meanings of the steps are:

- $s_{\mathrm{read}}$: read the current temperature;
- $s_{\mathrm{eval}}$: compare the temperature to the threshold;
- $s_{\mathrm{act}}$: switch the heating according to the decision.

The step-dependency relation inside the action is

$$next_{\alpha_{\mathrm{temp}}}=\left\{\left(s_{\mathrm{read}},s_{\mathrm{eval}}\right),\left(s_{\mathrm{eval}},s_{\mathrm{act}}\right)\right\}.$$

The first executable step is therefore $F_{\alpha_{\mathrm{temp}}}=\left\{s_{\mathrm{read}}\right\}$.

Hence, the action is

$$\alpha_{\mathrm{temp}}=\left(S_{\alpha_{\mathrm{temp}}},F_{\alpha_{\mathrm{temp}}},next_{\alpha_{\mathrm{temp}}}\right).$$

The complete step set of the recipe is $S=S_{\alpha_{\mathrm{temp}}}$.

The predecessor function is $pre(s_{\mathrm{read}})=⌀$, $pre\left(s_{\mathrm{eval}}\right)=\left\{s_{\mathrm{read}}\right\}$, and $pre\left(s_{\mathrm{act}}\right)=\left\{s_{\mathrm{eval}}\right\}$.

Data dependencies.

The only session parameter is the threshold, $\rho =(\rho_{\mathrm{thr}})$ with $\rho_{\mathrm{thr}}=23$. The data dependencies are

$$dep\left(s_{\mathrm{read}}\right)=⌀,\;dep\left(s_{\mathrm{eval}}\right)=\left\{s_{\mathrm{read}}\right\},\;dep\left(s_{\mathrm{act}}\right)=\left\{s_{\mathrm{eval}}\right\},$$

which satisfies $dep\left(s\right)\subseteq pre^{+}\left(s\right)$, and the input-composition functions are

$$\kappa_{s_{\mathrm{read}}}\left(\rho \right)=\left(\;\right),\;\kappa_{s_{\mathrm{eval}}}\left(\rho ,r\right)=\left(r\left(s_{\mathrm{read}}\right),\;\rho_{\mathrm{thr}}\right),\;\kappa_{s_{\mathrm{act}}}\left(\rho ,r\right)=r\left(s_{\mathrm{eval}}\right).$$

The service requirement function θ:S→Θ maps the steps to the required Arrowhead service definitions: $\theta \left(s_{\mathrm{read}}\right)=\theta_{\mathrm{read}}$, $\theta \left(s_{\mathrm{eval}}\right)=\theta_{\mathrm{eval}}$, and $\theta \left(s_{\mathrm{act}}\right)=\theta_{\mathrm{act}}$.

For simplicity, assume a common interface, no additional metadata constraints, and no QoS constraints: $\iota \left(s\right)=\iota_{0},\;\;\mu \left(s\right)=⌀,\;\;q\left(s\right)=⌀,\;\;s\in S$.

No special workflow executor capability is required in this example: η(s) is undefined for every s∈S.

Each step is executed once: qnty(s)=1,s∈S.

The complete workflow recipe is therefore W=(P,A,S,θ,ι,μ,q,pre,η,qnty,dep,κ).

### 7.3. Initial Workflow Session State

Let $z_{0}\in Z_{i}$ denote the initial state of one workflow session of recipe *W* in Local Cloud $LC_{i}$. We write $M_{k}$, $\beta_{k}$, $\xi_{k}$, and $status_{k}$ for the step-marking component, service-binding component, execution-context component, and session-status component of state $z_{k}$, respectively.

Initially, no step has been executed and no service binding has been selected: $M_{0}\left(s\right)=\mathrm{inactive},\;\;\beta_{0}\left(s\right)=⊥,\;\;s\in S$.

The execution context is initially empty: $\xi_{0}\left(s\right)$ is undefined for every s∈S.

The workflow session is running: $status_{0}=\mathrm{running}$.

The only action is active in the initial state, $M_{A,0}\left(\alpha_{\mathrm{temp}}\right)=\mathrm{active}$.

Since $pre(s_{\mathrm{read}})=⌀$, the first step that may become enabled is $s_{\mathrm{read}}$.

### 7.4. Reading the Temperature

The first step becomes enabled: $M_{1}\left(s_{\mathrm{read}}\right)=\mathrm{enabled}$. Transition $z_{0}\overset{\mathrm{enable}(s_{\mathrm{read}})}{\to }z_{1}$ (rule S-Enable).

The workflow choreographer compiles the step into an Arrowhead orchestration request: $req_{1}=compile_{i}\left(s_{\mathrm{read}},z_{1}\right)$.

Since no dedicated workflow executor is required, the consumer of the service is the workflow choreographer: $c_{z_{1}}\left(s_{\mathrm{read}}\right)=CH_{i}$.

At time $t_{1}$, the Orchestrator selects a feasible binding for the temperature sensor service:

$$O_{i}\left(c_{z_{1}}\left(s_{\mathrm{read}}\right),req_{1},\gamma_{s_{\mathrm{read}},z_{1}},t_{1}\right)=\pi_{1},\;\pi_{1}\in \Pi_{i}.$$

The selected binding is stored in the workflow session state: $\beta_{2}\left(s_{\mathrm{read}}\right)=\pi_{1}$. This is the transition $z_{1}\overset{\mathrm{bind}(s_{\mathrm{read}},\pi_{1})}{\to }z_{2}$ (rule S-Bind); it also sets $M_{2}\left(s_{\mathrm{read}}\right)=\mathrm{running}$.

The service is invoked with empty input data: $d_{1}=input\left(s_{\mathrm{read}},z_{2}\right)=\kappa_{s_{\mathrm{read}}}\left(\rho \right)=\left(\;\right)$.

Assume that the sensor returns the value $consume_{i}\left(\pi_{1},d_{1}\right)=24.6$.

The measured temperature is stored in the execution context: $\xi_{3}\left(s_{\mathrm{read}}\right)=24.6$.

After the successful invocation, the step completes: $M_{3}\left(s_{\mathrm{read}}\right)=\mathrm{completed}$. This is the transition $z_{2}\overset{\mathrm{done}(s_{\mathrm{read}})}{\to }z_{3}$ (rule S-Done); the result stored above is part of the same transition.

### 7.5. Evaluating the Temperature

The second step can execute after its predecessor has completed. Since $pre\left(s_{\mathrm{eval}}\right)=\left\{s_{\mathrm{read}}\right\}$ and $M_{3}\left(s_{\mathrm{read}}\right)=\mathrm{completed}$, the step $s_{\mathrm{eval}}$ becomes enabled: $M_{4}\left(s_{\mathrm{eval}}\right)=\mathrm{enabled}$. Transition $z_{3}\overset{\mathrm{enable}(s_{\mathrm{eval}})}{\to }z_{4}$ (rule S-Enable).

The step is compiled into an orchestration request: $req_{2}=compile_{i}\left(s_{\mathrm{eval}},z_{4}\right)$.

At time $t_{2}$, the Orchestrator selects a feasible binding for the temperature evaluation service:

$$O_{i}\left(c_{z_{4}}\left(s_{\mathrm{eval}}\right),req_{2},\gamma_{s_{\mathrm{eval}},z_{4}},t_{2}\right)=\pi_{2},\;\pi_{2}\in \Pi_{i}.$$

The selected binding is stored as $\beta_{5}\left(s_{\mathrm{eval}}\right)=\pi_{2}$. This is the transition $z_{4}\overset{\mathrm{bind}(s_{\mathrm{eval}},\pi_{2})}{\to }z_{5}$ (rule S-Bind); it also sets $M_{5}\left(s_{\mathrm{eval}}\right)=\mathrm{running}$.

The input of the evaluation step is constructed from the current workflow session state. In particular, it uses the value produced by $s_{\mathrm{read}}$:

$$d_{2}=input\left(s_{\mathrm{eval}},z_{5}\right)=\kappa_{s_{\mathrm{eval}}}\left(\rho ,\xi_{5}{|}_{dep(s_{\mathrm{eval}})}\right)=\left(\xi_{3}\left(s_{\mathrm{read}}\right),23\right).$$

Since $\xi_{3}\left(s_{\mathrm{read}}\right)=24.6$ and 24.6>23, the evaluation service returns the decision that the heating should be switched off: $consume_{i}\left(\pi_{2},d_{2}\right)=\mathrm{true}$.

This result is stored in the execution context: $\xi_{6}\left(s_{\mathrm{eval}}\right)=\mathrm{true}$.

The evaluation step then completes: $M_{6}\left(s_{\mathrm{eval}}\right)=\mathrm{completed}$. This is the transition $z_{5}\overset{\mathrm{done}(s_{\mathrm{eval}})}{\to }z_{6}$ (rule S-Done); the result stored above is part of the same transition.

### 7.6. Switching the Heating

The final step can execute after the evaluation step has completed. Since $pre\left(s_{\mathrm{act}}\right)=\left\{s_{\mathrm{eval}}\right\}$ and $M_{6}\left(s_{\mathrm{eval}}\right)=\mathrm{completed}$, the step $s_{\mathrm{act}}$ becomes enabled: $M_{7}\left(s_{\mathrm{act}}\right)=\mathrm{enabled}$. Transition $z_{6}\overset{\mathrm{enable}(s_{\mathrm{act}})}{\to }z_{7}$ (rule S-Enable).

The step is compiled into an Arrowhead orchestration request: $req_{3}=compile_{i}\left(s_{\mathrm{act}},z_{7}\right)$.

At time $t_{3}$, the Orchestrator selects a feasible binding for the heating switch service:

$$O_{i}\left(c_{z_{7}}\left(s_{\mathrm{act}}\right),req_{3},\gamma_{s_{\mathrm{act}},z_{7}},t_{3}\right)=\pi_{3},\;\pi_{3}\in \Pi_{i}.$$

The selected binding is stored in the workflow session state: $\beta_{8}\left(s_{\mathrm{act}}\right)=\pi_{3}$. This is the transition $z_{7}\overset{\mathrm{bind}(s_{\mathrm{act}},\pi_{3})}{\to }z_{8}$ (rule S-Bind); it also sets $M_{8}\left(s_{\mathrm{act}}\right)=\mathrm{running}$.

The input of the switching step is constructed from the evaluation result:

$$d_{3}=input\left(s_{\mathrm{act}},z_{8}\right)=\kappa_{s_{\mathrm{act}}}\left(\rho ,\xi_{8}{|}_{dep(s_{\mathrm{act}})}\right)=\xi_{6}\left(s_{\mathrm{eval}}\right).$$

The heating switch service is invoked: $consume_{i}\left(\pi_{3},d_{3}\right)=\mathrm{OFF}$.

The resulting switch state is stored in the execution context: $\xi_{9}\left(s_{\mathrm{act}}\right)=\mathrm{OFF}$.

After the successful invocation, the switching step completes: $M_{9}\left(s_{\mathrm{act}}\right)=\mathrm{completed}$. This is the transition $z_{8}\overset{\mathrm{done}(s_{\mathrm{act}})}{\to }z_{9}$ (rule S-Done); the result stored above is part of the same transition.

**Figure 7:**
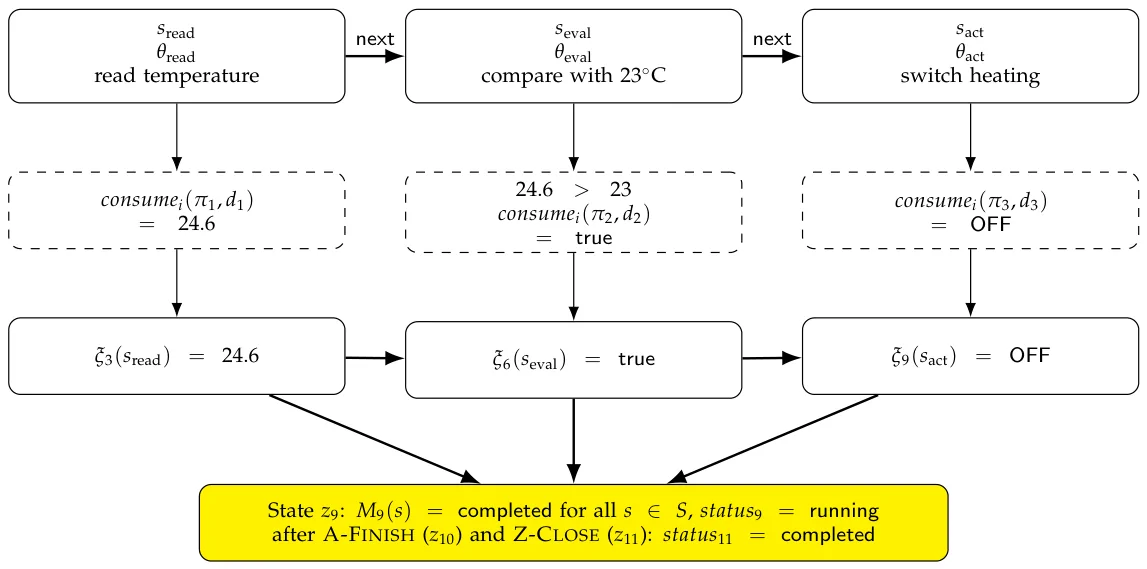
Concrete execution trace of the temperature-control demonstration. The sensor value 24.6 is stored in the execution context, the evaluation step derives the decision true, and the heating-switch step stores the final state OFF. After $z_{9}$, the action finishes ($z_{10}$) and the session closes ($z_{11}$).

Figure 7 visualizes the three steps of the recipe and the execution of the workflow, as in the generic example, but with concrete services and values.

### 7.7. Final Workflow Session State

At the end of the workflow session, all steps have completed: $M_{9}\left(s\right)=\mathrm{completed},\;s\in S$.

The final execution-context values are $\xi_{9}\left(s_{\mathrm{read}}\right)=24.6$, $\xi_{9}\left(s_{\mathrm{eval}}\right)=\mathrm{true}$, and $\xi_{9}\left(s_{\mathrm{act}}\right)=\mathrm{OFF}$.

After $z_{9}$, all steps have completed, $M_{9}\left(s\right)=\mathrm{completed}$ for s∈S, but the session is still running, $status_{9}=\mathrm{running}$. The action and the session are then closed by the rules A-Finish and Z-Close: $M_{A,10}\left(\alpha_{\mathrm{temp}}\right)=\mathrm{completed}$ and $status_{11}=\mathrm{completed}$. The complete run, in which every arrow is an instance of the indicated rule of Section 5.9, is

$$\begin{array}{cc} z_{0} & \overset{\mathrm{enable}(s_{\mathrm{read}})}{\to }z_{1}\overset{\mathrm{bind}(s_{\mathrm{read}},\pi_{1})}{\to }z_{2}\overset{\mathrm{done}(s_{\mathrm{read}})}{\to }z_{3}\\ & \overset{\mathrm{enable}(s_{\mathrm{eval}})}{\to }z_{4}\overset{\mathrm{bind}(s_{\mathrm{eval}},\pi_{2})}{\to }z_{5}\overset{\mathrm{done}(s_{\mathrm{eval}})}{\to }z_{6}\\ & \overset{\mathrm{enable}(s_{\mathrm{act}})}{\to }z_{7}\overset{\mathrm{bind}(s_{\mathrm{act}},\pi_{3})}{\to }z_{8}\overset{\mathrm{done}(s_{\mathrm{act}})}{\to }z_{9}\\ & \overset{\mathrm{finish}(\alpha_{\mathrm{temp}})}{\to }z_{10}\overset{\mathrm{close}}{\to }z_{11}. \end{array}$$

This run shows how the workflow recipe is executed by repeatedly compiling steps into Arrowhead orchestration requests, obtaining service bindings from the Orchestrator, invoking the selected services, and storing the resulting data in the workflow session context. Table 8 lists it state by state. Table 8 is not an experimental result. It lists one execution of the temperature-control recipe, so that every transition can be checked against the rules of Section 5.9. The last column relates each state change to the corresponding event in the plant: the reading of 24.6 °C, the decision to switch the heating off because 24.6>23, and the confirmation that the switch is OFF. The formal marking and binding values of each state are given in the preceding subsections.

**Table 8:** Execution trace of the temperature-control demonstration: the transition rule applied in each state and the corresponding event in the plant.

State	Rule	Event in the Plant
$z_{0}$	initial state	Session created for one control period; nothing has been executed yet.
$z_{1}$	S-Enable $\left(s_{\mathrm{read}}\right)$	The measurement cycle starts: reading the temperature is allowed.
$z_{2}$	S-Bind $\left(s_{\mathrm{read}},\pi_{1}\right)$	The Orchestrator finds and authorizes the temperature sensor service.
$z_{3}$	S-Done $\left(s_{\mathrm{read}}\right)$	The value 24.6 °C is measured and stored in ξ.
$z_{4}$	S-Enable $\left(s_{\mathrm{eval}}\right)$	Evaluation is allowed, because the reading has completed.
$z_{5}$	S-Bind $\left(s_{\mathrm{eval}},\pi_{2}\right)$	The evaluation service of the controller is found and authorized.
$z_{6}$	S-Done $\left(s_{\mathrm{eval}}\right)$	As 24.6>23, the decision true (switch the heating off) is stored.
$z_{7}$	S-Enable $\left(s_{\mathrm{act}}\right)$	Actuation is allowed only now, after the evaluation has completed.
$z_{8}$	S-Bind $\left(s_{\mathrm{act}},\pi_{3}\right)$	The heating-switch service is found and authorized.
$z_{9}$	S-Done $\left(s_{\mathrm{act}}\right)$	The switch reports OFF, which is stored.
$z_{10}$	A-Finish $\left(\alpha_{\mathrm{temp}}\right)$	All steps of the only action have completed.
$z_{11}$	Z-Close	The control period is closed; the session is completed.

## 8. Worked Example—Shared Tool Delivery in a Forklift Assembly Plant

The previous examples use a single action and a linear chain of three steps. This example is closer to an industrial setting and exercises the remaining features of the model: several actions, parallel steps, data dependencies across actions, delegation of steps to a workflow executor, and several concurrent sessions of one recipe. An interactive simulation of this example, with three concurrent line sessions and injectable failures, is available at https://arrowhead-workflows.vercel.app/forklift [42] (accessed on 2 November 2026).

A forklift manufacturer operates three assembly lines, $L_{1}$, $L_{2}$ and $L_{3}$. Some forklift variants contain a part whose fasteners must be tightened with a specialized nutrunner. The tool is expensive and needed only for these variants, and only occasionally, so a single tool is shared by the three lines. It is stored in a tool crib. When the line controller of $L_{k}$ recognizes that the current variant requires the tool, it requests the service. The tool is then reserved, an automated guided vehicle (AGV) brings it to the station of $L_{k}$, the fasteners are tightened according to the tightening program of the variant, the result is checked and recorded, and the AGV returns the tool to the crib so that another line can use it. The decision of whether a variant needs the tool is taken by the line controller, which starts a workflow session only in that case. Conditional execution inside a recipe is therefore not needed and not modeled.

### 8.1. Application Systems and Assumptions

The Local Cloud $LC_{i}$ contains the application systems of Table 9, the workflow choreographer $CH_{i}$, and one workflow executor, $WE_{i}=\left\{w_{\mathrm{agv}}\right\}$, which supervises long-running AGV missions.

The example makes the following assumptions about these systems.

(A1) ReserveTool returns a reservation token *r* only when the tool is granted to the requester. If the tool is held by another session, the call stays pending until the holder has been released via ReleaseTool. Hence, at most one session holds the tool at any time, from the return of ReserveTool until ReleaseTool is processed.
(A2) Only $w_{\mathrm{agv}}$ is authorized to consume TransportTool. The two AGV steps are therefore delegated, with $\eta \left(s_{\mathrm{move}}\right)=\eta \left(s_{\mathrm{ret}}\right)=e_{\mathrm{agv}}$, where $e_{\mathrm{agv}}\in E$ describes the execution of AGV missions. The executor satisfies it, $cap\left(w_{\mathrm{agv}}\right)=\left\{e_{\mathrm{agv}}\right\}$; hence, $w_{\mathrm{agv}}⊧e_{\mathrm{agv}}$, and it is the only candidate. A session in which $w_{\mathrm{agv}}$ is offline has asg(z,s)=⌀ for the AGV steps and therefore ends with S-FailBind.
(A3) The provider instance of ConfigureTool and TightenFasteners is the controller of the shared nutrunner. It is selected through the session-dependent request parameter λ(s,z), which carries the tool identifier $\rho_{\mathrm{tool}}$.

### 8.2. Workflow Recipe Instance

**Figure 8:**
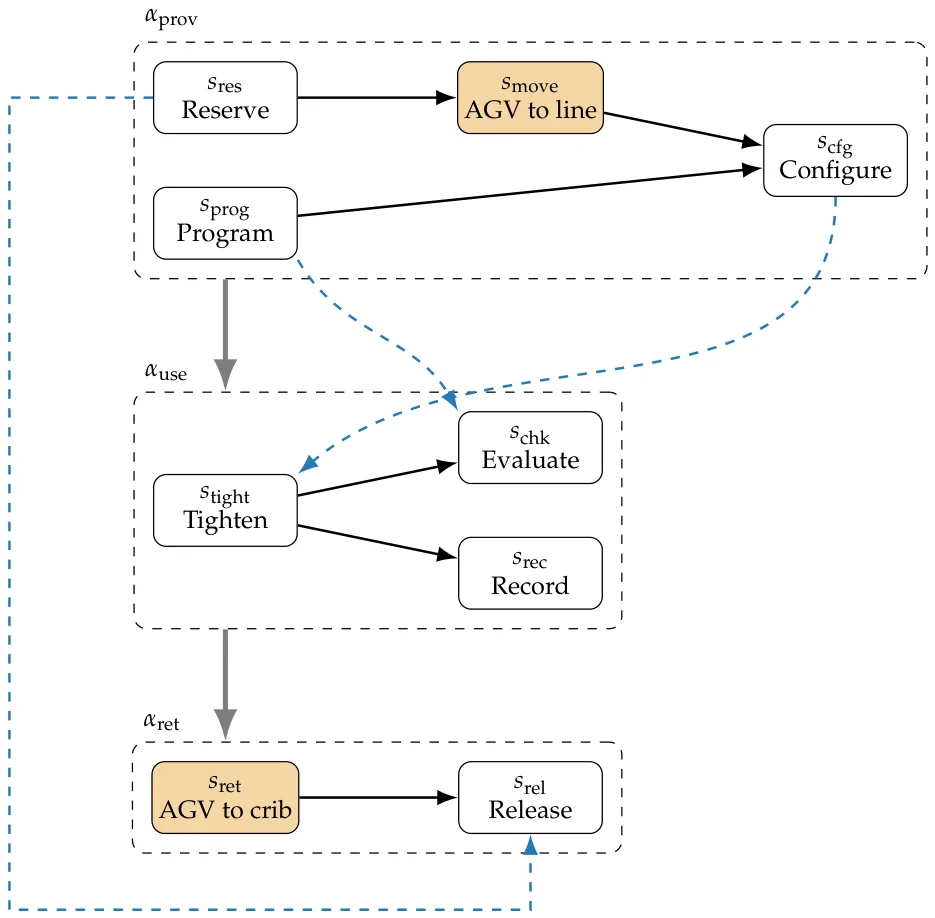
Workflow recipe of the shared-tool example. Solid thin arrows: step dependencies ${\mathrm{next}}_{\alpha }$ inside an action. Thick gray arrows: action order ${\mathrm{next}}_{A}$. Dashed blue arrows: data dependencies dep that cross actions. Orange steps are delegated to the workflow executor $w_{\mathrm{agv}}$ (η defined). The dependencies of $s_{\mathrm{move}}$ on $s_{\mathrm{res}}$ and of $s_{\mathrm{chk}}$ on $s_{\mathrm{tight}}$, and the $\kappa_{s}$ inputs, are listed in Table 10.

The recipe has one plan with three actions, which provision the tool, use it, and return it:

$$A=\left\{\alpha_{\mathrm{prov}},\alpha_{\mathrm{use}},\alpha_{\mathrm{ret}}\right\},\;\alpha_{0}=\alpha_{\mathrm{prov}},\;next_{A}=\left\{\left(\alpha_{\mathrm{prov}},\alpha_{\mathrm{use}}\right),\left(\alpha_{\mathrm{use}},\alpha_{\mathrm{ret}}\right)\right\},$$

and $P=(\mathrm{SharedToolTightening},A,\alpha_{0},next_{A})$. The step sets and step dependencies of the actions are

$$\begin{array}{cccc} S_{\mathrm{prov}} & =\{s_{\mathrm{res}},s_{\mathrm{prog}},s_{\mathrm{move}},s_{\mathrm{cfg}}\}, & next_{\mathrm{prov}} & =\{\left(s_{\mathrm{res}},s_{\mathrm{move}}\right),\left(s_{\mathrm{move}},s_{\mathrm{cfg}}\right),\left(s_{\mathrm{prog}},s_{\mathrm{cfg}}\right)\},\\ S_{\mathrm{use}} & =\{s_{\mathrm{tight}},s_{\mathrm{chk}},s_{\mathrm{rec}}\}, & next_{\mathrm{use}} & =\{\left(s_{\mathrm{tight}},s_{\mathrm{chk}}\right),\left(s_{\mathrm{tight}},s_{\mathrm{rec}}\right)\},\\ S_{\mathrm{ret}} & =\{s_{\mathrm{ret}},s_{\mathrm{rel}}\}, & next_{\mathrm{ret}} & =\{\left(s_{\mathrm{ret}},s_{\mathrm{rel}}\right)\}, \end{array}$$

so that |S|=9, $F_{\mathrm{prov}}=\left\{s_{\mathrm{res}},s_{\mathrm{prog}}\right\}$, $F_{\mathrm{use}}=\left\{s_{\mathrm{tight}}\right\}$ and $F_{\mathrm{ret}}=\left\{s_{\mathrm{ret}}\right\}$. Figure 8 shows the recipe. The steps are: reserve the tool ($s_{\mathrm{res}}$), obtain the tightening program of the variant ($s_{\mathrm{prog}}$), move the tool to the station ($s_{\mathrm{move}}$), load the program into the tool ($s_{\mathrm{cfg}}$), tighten ($s_{\mathrm{tight}}$), evaluate the result ($s_{\mathrm{chk}}$), record it ($s_{\mathrm{rec}}$), move the tool back to the crib ($s_{\mathrm{ret}}$), and release the reservation ($s_{\mathrm{rel}}$).

The remaining components of the recipe are given in Table 10. All steps use the same interface, $\iota \left(s\right)=\iota_{0}$, have no metadata or QoS requirements, μ(s)=q(s)=⌀, and are executed once, qnty(s)=1. In particular the tightening of all bolts is a single invocation of TightenFasteners, which the tool controller executes according to the program, so $qnty(s_{\mathrm{tight}})=1$. The session parameters are $\rho =(\rho_{\mathrm{tool}},\rho_{\mathrm{line}},\rho_{\mathrm{stn}},\rho_{\mathrm{var}},\rho_{\mathrm{crib}})$: tool identifier, requesting line, station of that line, forklift variant, and crib location. A tightening program is a triple $\left(n,T^{*},\delta \right)$ of bolt count, target torque and relative tolerance. For $r\in D^{dep(s)}$, $r(s^{\prime })$ denotes the result of step $s^{\prime }$.

The recipe is well-formed. The action graph is the path $\alpha_{\mathrm{prov}}\to \alpha_{\mathrm{use}}\to \alpha_{\mathrm{ret}}$, and the step graphs are finite and acyclic. Every data dependency is allowed by (W4). Some follow from pre, for example, $s_{\mathrm{res}}\in pre\left(s_{\mathrm{move}}\right)$. The others cross actions, for example, $s_{\mathrm{cfg}}≺s_{\mathrm{tight}}$ and $s_{\mathrm{res}}≺s_{\mathrm{rel}}$, because $\alpha_{\mathrm{prov}}$ precedes $\alpha_{\mathrm{use}}$ and $\alpha_{\mathrm{ret}}$ in $G_{A}$; here, $s_{\mathrm{cfg}}\notin pre^{+}\left(s_{\mathrm{tight}}\right)$. Control and data dependencies also differ inside an action: $s_{\mathrm{move}}\in pre\left(s_{\mathrm{cfg}}\right)$, since the tool must be at the station before it is configured, but $s_{\mathrm{move}}\notin dep\left(s_{\mathrm{cfg}}\right)$, since its result is not needed as input. The same service definition TransportTool is required by two steps, which differ only through $\kappa_{s}$ (crib to station, and station to crib).

### 8.3. Execution of One Session

Line $L_{2}$ needs the tool for a variant *v*. Its controller starts a session with $\rho_{2}=\left(\mathrm{NR}7,2,{\mathrm{st}}_{2},v,\mathrm{crib}\right)$. The initial state $z_{0}$ is as defined in Section 5.3: all steps are inactive, and $M_{A,0}\left(\alpha_{\mathrm{prov}}\right)=\mathrm{active}$. Since |S|=9 and |A|=3, every failure-free run of this session has exactly 3|S|+2|A|=33 transitions, whatever the interleaving of the parallel steps. Table 11 lists one such run in phases, for illustrative data values.

Delegation.

In $z_{7}$ (phase 3), $s_{\mathrm{move}}$ is enabled. Since $\eta \left(s_{\mathrm{move}}\right)=e_{\mathrm{agv}}$, rule S-Bind assigns the executor: $asg\left(z_{7},s_{\mathrm{move}}\right)=\left\{z^{\prime }\right\}$ with $z^{\prime }=z_{7}\left[\varepsilon \left(s_{\mathrm{move}}\right)\mapsto w_{\mathrm{agv}}\right]$, so that $c_{z^{\prime }}\left(s_{\mathrm{move}}\right)=w_{\mathrm{agv}}$. By (A2), the choreographer itself would not obtain a binding, whereas the executor does:

$$\begin{array}{cc} O_{i}\left(CH_{i},\;compile_{i}\left(s_{\mathrm{move}},z^{\prime }\right),\;\gamma_{s_{\mathrm{move}},z^{\prime }},\;t\right) & =⊥,\\ O_{i}\left(w_{\mathrm{agv}},\;compile_{i}\left(s_{\mathrm{move}},z^{\prime }\right),\;\gamma_{s_{\mathrm{move}},z^{\prime }},\;t\right) & =\pi_{\mathrm{move}}. \end{array}$$

The executor then invokes the selected service, and the step completes when the mission report is returned.

Data composition across actions.

After $s_{\mathrm{tight}}$ has stored the tightening results $R=(T_{1},\ldots ,T_{8})$ in ξ, the input of $s_{\mathrm{chk}}$ combines this result with the program stored by $s_{\mathrm{prog}}$ in the first action:

$$input\left(s_{\mathrm{chk}},z\right)=\kappa_{s_{\mathrm{chk}}}\left(\rho_{2},\xi_{z}{|}_{dep(s_{\mathrm{chk}})}\right)=\left(\xi_{z}\left(s_{\mathrm{tight}}\right),\;\xi_{z}\left(s_{\mathrm{prog}}\right)\right)=\left(R,\;(8,\;210\;N\cdot m,\;5\%)\right),$$

which is defined by Lemma 2, although $s_{\mathrm{prog}}$ is not a predecessor of $s_{\mathrm{chk}}$ in pre. The service EvaluateTightening returns the verdict OK when every $T_{j}$ lies within ±5% of 210N·m.

### 8.4. Concurrent Sessions and Exclusive Tool Use

The recipe is immutable, and each request creates a separate session with its own state. Suppose that $L_{1}$ requests the tool while the session $S_{2}$ of $L_{2}$ is in progress. Table 12 shows how the two sessions interleave. The session $S_{1}$ of $L_{1}$ has $M(s_{\mathrm{res}})=\mathrm{running}$ while it waits, because the call to ReserveTool is pending. Meanwhile, $s_{\mathrm{prog}}$ of $S_{1}$ proceeds, since it does not depend on the tool, but $s_{\mathrm{move}}$ stays inactive, because $s_{\mathrm{res}}\in pre\left(s_{\mathrm{move}}\right)$ is not completed.

**Table 12:** Interleaving of the sessions $S_{2}$ (line $L_{2}$) and $S_{1}$ (line $L_{1}$).

#	Session $S_{2}$	Session $S_{1}$	Tool
1	start; $s_{\mathrm{res}}$ bound, done	—	held by $S_{2}$
2	$s_{\mathrm{move}}$ running (AGV driving)	start; $s_{\mathrm{res}}$ bound, $M(s_{\mathrm{res}})=\mathrm{running}$	held by $S_{2}$
3	$s_{\mathrm{cfg}}$, $s_{\mathrm{tight}}$, $s_{\mathrm{chk}}$, $s_{\mathrm{rec}}$ done	$s_{\mathrm{prog}}$ done; $s_{\mathrm{move}}$ inactive	held by $S_{2}$
4	$s_{\mathrm{ret}}$ done; $s_{\mathrm{rel}}$ enabled	waiting	held by $S_{2}$
5	$s_{\mathrm{rel}}$ bound; ReleaseTool processed; done	—	free
6	finish, close: completed	ReserveTool returns; $s_{\mathrm{res}}$ done, $\xi \left(s_{\mathrm{res}}\right)=r_{1}$	held by $S_{1}$
7	—	$s_{\mathrm{move}}$ enabled; continues as in Table 11	held by $S_{1}$

The proposition refers to (A1), the original assumption; the lease variant is discussed below.

Mutual exclusion of the tool is provided by the tool manager (A1), not by the choreographer. What the recipe contributes is that, within each session, all uses of the tool lie inside the interval in which the session holds it.

**Proposition** **1** (Exclusive tool use)**.** *Let $U=\{s_{\mathrm{move}},s_{\mathrm{cfg}},s_{\mathrm{tight}},s_{\mathrm{ret}}\}$ be the steps that use the tool physically. Under (A1), the running intervals of steps of U belonging to different sessions are disjoint.*

**Proof.**  Consider one session. Let s∈U and let *s* leave inactive. Then, $s_{\mathrm{res}}≺s$: for $s_{\mathrm{move}}$ because $s_{\mathrm{res}}\in pre\left(s_{\mathrm{move}}\right)$, for $s_{\mathrm{cfg}}$ because $s_{\mathrm{res}}\in pre^{+}\left(s_{\mathrm{cfg}}\right)$, and for $s_{\mathrm{tight}}$ and $s_{\mathrm{ret}}$ because they belong to later actions than $s_{\mathrm{res}}$. By Lemma 1(iv), $s_{\mathrm{res}}$ is completed, so every step of *U* starts running after the tool has been granted. Conversely, when $s_{\mathrm{rel}}$ leaves inactive, all steps of *U* are completed: $s_{\mathrm{ret}}\in pre\left(s_{\mathrm{rel}}\right)$, and the other three belong to earlier actions. The invocation of ReleaseTool occurs while $s_{\mathrm{rel}}$ is running, and hence after every step of *U* has completed. Thus, all running intervals of steps of *U* lie inside the interval in which the session holds the tool, and these holding intervals are disjoint for different sessions by (A1). □

In relation to the features exercised, the example uses three actions, a step graph with two initial steps and a join ($s_{\mathrm{cfg}}$), parallel steps ($s_{\mathrm{chk}}\|s_{\mathrm{rec}}$), control and data dependencies that differ, data dependencies across actions, one service definition required by two steps, executor delegation with consumer-specific authorization, the marking running as a waiting state, three concurrent sessions of one immutable recipe, and a failure case.

The model also exposes a limit of the current failure semantics. If the AGV mission of $s_{\mathrm{move}}$ fails, for example, because the route is blocked, rule S-FailInvoke sets status=failed. At that point $s_{\mathrm{res}}$ is completed and $s_{\mathrm{rel}}$ is inactive, and no rule enables it. The reservation is never released, and the lines $L_{1}$ and $L_{3}$ wait forever. A compensating step that releases the tool after a failure requires the recovery semantics that is left for future work.

The failure can be written as a run. Suppose that in the session of line $L_{2}$, the AGV mission of $s_{\mathrm{move}}$ fails after $s_{\mathrm{res}}$ has completed. The run ends with the rule instance $z\overset{S-FailInvoke(s_{\mathrm{move}})}{\to }z^{\prime }$, where $M^{\prime }\left(s_{\mathrm{move}}\right)=\mathrm{running}$, $M^{\prime }\left(s_{\mathrm{res}}\right)=\mathrm{completed}$ and $status^{\prime }=\mathrm{failed}$. No rule applies in $z^{\prime }$, because the premises of all rules require status=running, and Theorem 2 covers only that case. Since $s_{\mathrm{move}}$ never completes, $s_{\mathrm{cfg}}$ and all steps of the later actions, in particular $s_{\mathrm{rel}}$, stay inactive, so the reservation made by $s_{\mathrm{res}}$ is never released. The step $s_{\mathrm{prog}}$ may be completed or not, depending on the interleaving.

The wait in (A1) also touches the assumption of Theorem 2: the pending call of ReserveTool of $L_{1}$ does not return while the failed holder has not released the tool. A variant of (A1) in which $AS_{\mathrm{crib}}$ grants leases removes this wait: a reservation that is neither renewed nor released expires, and the next queued call of ReserveTool is granted. In this variant, the sessions of $L_{1}$ and $L_{3}$ complete after the lease of the failed session has expired, and the failed session stays failed. The model needs no new rule for this, because the expiry is an event of $AS_{\mathrm{crib}}$ and not a transition of a session. The simulation offers the variant as an option, and the kit implements it: a tool-using step renews the lease with the reservation token $\xi (s_{\mathrm{res}})$ before every physical use and fails without a valid lease. For this renewal, the kit adds $s_{\mathrm{res}}$ to dep(s) of $s_{\mathrm{cfg}}$, $s_{\mathrm{tight}}$ and $s_{\mathrm{ret}}$. Condition (W4) still holds, because $s_{\mathrm{res}}≺s$ for these steps.

Proposition 1 is proved under (A1) only; it has not been proved for the lease variant. If a lease expires while the AGV is still moving the tool, the tool manager may grant the tool to the next queued session, and the model does not prevent two sessions from using the tool at the same time. The kit avoids this only by configuration: the lease is renewed before every physical use and is chosen longer than the longest single tool step. If this holds, i.e., if every step of *U* runs inside a lease that was renewed immediately before the step started, and the leases of different sessions are disjoint, the proof of Proposition 1 carries over with “holding interval” replaced by “lease interval”. We state this as an additional assumption and have not verified it formally.

## 9. Conclusions

This paper addressed a gap left open by the mathematical model of the Arrowhead Framework’s core systems, recalled in Section 3: while that model rigorously characterizes the execution of individual service interactions—service publication, discovery, authorization, and orchestration-based provider selection—it does not describe how multiple, dependent service interactions are coordinated into an executable workflow. Workflow choreography, although already available as a practical mechanism within the Eclipse Arrowhead project, had so far been described only operationally. This paper closed that gap by giving workflow choreography a compact, self-contained mathematical semantics that is conservative with respect to the existing Arrowhead formalism.

The static structure of a workflow was captured by the *Workflow Recipe*, organized as the three-level hierarchy Plan⊃Actions⊃Steps. The plan fixes the sequential ordering of actions through the transition relation $next_{A}$; each action contains a finite, acyclic step graph $G_{\alpha }$ in which parallel execution is permitted; and each step s∈S, with requirement profile σ(s)=(θ(s),ι(s),μ(s),q(s),pre(s),η(s),qnty(s)), specifies only the service requirements of one logical interaction, leaving concrete provider selection entirely to the Arrowhead Orchestrator. Because a workflow recipe contains no execution state, service bindings, or intermediate results, it remains immutable across arbitrarily many executions.

Runtime behavior was captured separately by the *Workflow Session* state $z=(M,M_{A},\beta ,\varepsilon ,\xi ,status)$, together with a compact execution model built around a single *fundamental execution primitive*: an enabled step is compiled into an ordinary Arrowhead request via $compile_{i}$, submitted to the orchestration function $O_{i}$ already recalled in Section 3, invoked through the abstract consumption function $consume_{i}$, and its result written back into the session context ξ, which in turn enables successor steps. Every step of this pipeline—compilation, orchestration, invocation, state update—was given an explicit mathematical definition, and the model additionally accounts for optional delegation of step execution to dedicated workflow executors through the capability function η and its runtime realization ε. Three worked examples, a generic monitoring-and-control workflow, a concrete temperature-control demonstration, and a shared-tool delivery workflow of a forklift assembly plant with parallel steps, executor delegation and concurrent sessions, showed that the notation is not merely descriptive but directly instantiable: every symbol introduced in the general model was given a concrete value, and the resulting state trace $z_{0}\to z_{1}\to \cdots \to z_{11}$ reproduced, step by step, the behavior expected of the Arrowhead workflow choreographer. The examples are also available as executable demonstrators, and both were executed on the workflow choreographer of Eclipse Arrowhead release 4.6.2 (Section 5.12).

The central property established throughout the paper is that workflow choreography contributes coordination semantics only. It introduces no alternative service-discovery, authorization, QoS-filtering, or provider-selection mechanism: every service invocation triggered during workflow execution is still governed exclusively by the Arrowhead Orchestrator $O_{i}$, and workflow choreography merely determines which step becomes enabled next and how its request is assembled. This makes the proposed model a strictly conservative extension of the Arrowhead Framework—one that can be adopted without modifying the Service Registry, authorization system, Orchestrator, gatekeeper, or gateway, and without weakening any guarantee already established for individual service interactions by the Arrowhead Framework [8,9].

The present formalization deliberately kept its scope narrow so that the core execution semantics could be stated compactly. The scope of the results is as follows. Termination and deadlock-freedom of a workflow session are proved for the transition system of Section 5.9 (Theorems 1 and 2), but only for runs without recovery and with qnty=1. The examples exercise multiple actions, parallel steps, workflow executor delegation, data flow across actions, concurrent sessions and one failure case. Interactive simulations of both worked examples [42] apply the rules of Section 5.9 and check, on the executed runs, Lemma 2, the bound of Theorem 1, progress and, for the tool delivery example, Proposition 1.

As an important limitation, this work does not handle inter-cloud choreography. The model covers one Local Cloud, similarly to previous Arrowhead choreographer-related papers. Workflow choreography spanning multiple Local Clouds through the gatekeeper and gateway is excluded, and inherited unmodified from the standard Arrowhead Framework specification [8,9]. To address this as future work, we need to extend the model to steps whose providers reside in different Local Clouds, reusing the gatekeeper- and gateway-based mechanisms of the Arrowhead Framework.

Another important limitation is related to the current demonstrators: repeated execution is not shown, and the rules assume that qnty(s)=1. The session state holds one marking, one binding and one result per step, so a step cannot be executed repeatedly, and the examples do not cover this case. To handle this, as future work, we need to add execution counters to the workflow session state for qnty(s)>1, with the corresponding state updates, and extend Lemma 1 and the bound of Theorem 1. After that, we have to show its aligned demonstration.

Altogether, the model itself provides a specification against which the Arrowhead workflow choreographer implementation can be validated, and a foundation for further tooling, supporting the flexible, dynamically composable production workflows demanded by Industry 4.0 and Industry 5.0 applications.

## Figures and Tables

**Table 1:** Synthesis of related work on workflow management (Section 2.2, Section 2.3, Section 2.4, Section 2.5 and Section 2.6). NOTE: Column “FM?” states whether formal methods are addressed in the papers or not.

Section	Representative Works	Coordination Focus	FM?	Gap Relative to This Paper
Formal models of orchestration & choreography (Section 2.2)	Peltz [20] Zhao et al. [21] Lucchi et al. [22] van der Aalst [23,24] Dijkman et al. [25]	Give WS-CDL, WS-BPEL, Petri-net workflows, and BPMN a precise, checkable execution or control-flow semantics	Yes	Services are statically bound or resolved by an external registry, not re-orchestrated per execution as in Arrowhead
Foundations and surveys of workflow management (Section 2.3)	Georgakopoulos, Hornick & Sheth [27] van der Aalst [28]	Establish the process/case/task/resource vocabulary and life-cycle view (design, enactment, diagnosis) of workflow and BPM systems	Part.	Predate, and remain agnostic to, dynamically orchestrated service substrates such as Arrowhead
Workflow management for manufacturing (Section 2.4)	Hawaou et al. [29] Liu et al. [30] Rashidifar et al. [31] Kozma et al. [14]	Survey workflow/task scheduling—makespan, cost, energy, QoS—for shop-floor and cloud-manufacturing settings	No	Scheduling is optimized above an assumed-executable process model; how a scheduled task becomes a concrete service invocation is left unformalized
Workflow management for supply chains (Section 2.5)	Ben-Daya et al. [37] Frederico et al. [38] Zhang et al. [39] Wannenwetsch et al. [40] Kozma et al. [41]	Review IoT-driven, blockchain-based, and Industry-4.0-maturity approaches to cross-organizational process coordination	No	Focus on trust, traceability, and maturity assessment rather than on precise execution semantics for the coordinated process steps
Arrowhead-based workflow management (Section 2.6)	Kozma, Varga & Szabó [15] Kozma et al. [13,14] Garcia et al. [16]	Introduce and engineer the Arrowhead workflow choreographer: dynamic composition of steps into Arrowhead requests, BPMN/CPN and microservice-based alternatives	No	Describes what the choreographer does, not mathematical execution semantics tied to the orchestration function $O_{i}$

**Table 2:** Summary of symbols used for the Arrowhead core systems and service requests (Section 3).

Symbol	Definition
Arrowhead core systems and requests (recalled in Section 3)	
$LC_{i}$	Arrowhead Local Cloud indexed by *i*.
*T*	System-time domain.
t∈T	A point in system time.
${\mathrm{APP}}_{i}$	Set of application systems in $LC_{i}$.
$C_{i}^{min}$	Set of mandatory core systems of $LC_{i}$.
$C_{i}^{sup}$	Set of supporting core systems of $LC_{i}$ considered in this model.
${\mathrm{SYS}}_{i}$	Set of all systems in $LC_{i}$ ($={\mathrm{APP}}_{i}\cup C_{i}^{min}\cup C_{i}^{sup}$).
$\sigma_{i}$	Set of abstract service types known in $LC_{i}$.
$R_{i}$, $R_{i}\left(t\right)$	Service Registry of $LC_{i}$ (time-dependent).
$O_{i}$	Orchestrator of $LC_{i}$, formalized as the orchestration function $O_{i}:Cons_{i}\times Req_{i}\times \Gamma_{i}\times T\to \Pi_{i}\cup \left\{⊥\right\}$.
$A_{i}$	Authorization system of $LC_{i}$.
$GK_{i}$	Gatekeeper of $LC_{i}$ (supporting system).
$GW_{i}$	Gateway of $LC_{i}$ (supporting system).
$QM_{i}$	QoS manager of $LC_{i}$ (supporting system).
$Cons_{i}$	Set of systems that may act as service consumers in $LC_{i}$.
$Req_{i}$	Orchestration-request domain of $LC_{i}$.
$\Gamma_{i}$	Orchestration-constraint space of $LC_{i}$.
$\gamma_{s,z}$	Orchestration constraints applicable to step *s* in session state *z*.
$\Pi_{i}$	Set of feasible orchestration plans (service bindings) in $LC_{i}$.
$\pi \in \Pi_{i}$	A feasible orchestration plan, i.e., a selected service binding.
⊥	Failure symbol, or absent value (e.g., no binding, infeasible orchestration).

**Table 3:** Summary of symbols used in the workflow recipe (Section 4).

Symbol	Definition
Workflow Recipe	
*W*	Workflow recipe, W=(P,A,S,θ,ι,μ,q,pre,η,qnty,dep,κ).
*P*	Plan of a workflow recipe, $P=(name,A,\alpha_{0},next_{A})$.
name	Workflow identifier.
*A*	Finite action set of the plan.
$\alpha_{0}$	Initial action of the plan, $\alpha_{0}\in A$.
$next_{A}$	Action transition relation, $next_{A}\subseteq A\times A$.
$G_{A}=\left(A,E_{A}\right)$	Action graph induced by the plan ($E_{A}=next_{A}$).
α∈A	A workflow action, $\alpha =(S_{\alpha },F_{\alpha },next_{\alpha })$.
$S_{\alpha }$	Step set of action α.
$F_{\alpha }\subseteq S_{\alpha }$	Steps of action α without predecessors.
$next_{\alpha }$	Step-dependency relation inside action α, $next_{\alpha }\subseteq S_{\alpha }\times S_{\alpha }$.
$G_{\alpha }=\left(S_{\alpha },E_{\alpha }\right)$	Step graph of action α ($E_{\alpha }=next_{\alpha }$).
*S*	Complete step universe of the workflow recipe, $S={⋃}_{\alpha \in A}S_{\alpha }$.
s∈S	A workflow step (identifier) with requirement profile σ(s)=(θ(s),ι(s),μ(s),q(s),pre(s),η(s),qnty(s)).
θ	Service-definition requirement function, θ:S→Θ.
Θ	Service-definition domain.
ι	Required-interface function, ι:S→I.
*I*	Interface domain.
μ	Metadata-requirement function, μ:S→MD.
MD	Metadata domain.
*q*	QoS-requirement function, q:S→Q.
*Q*	QoS-requirement domain.
pre	Predecessor-step function, $pre:S\to 2^{S}$.
η	Optional workflow executor capability function, η:S⇀E.
*E*	Domain of workflow executor capability descriptions.
qnty	Step execution-multiplicity function, qnty:S→N.
dep	Data-dependency function, $dep:S\to 2^{S}$, with dep(s)⊆{p∣p≺s}.
$\kappa_{s}$	Input-composition function of step *s*, $\kappa_{s}:P\times D^{dep(s)}\to D$.
P	Domain of session parameters (constants fixed at session creation).
α(s)	The unique action containing step *s*.
$pre^{+}$	Transitive closure of pre.
p≺s	Step *p* precedes step *s*: $p\in pre^{+}\left(s\right)$, or the action of *p* precedes the action of *s* in $G_{A}$.

**Table 4:** Summary of symbols used for the formal workflow execution model.

Symbol	Definition
*Workflow Session and Execution Model (Section 5)*	
$CH_{i}\in Cons_{i}$	Workflow choreographer of $LC_{i}$.
$WE_{i}\subseteq Cons_{i}$	Set of workflow executors available to the choreography system.
cap	Capability set of a workflow executor, $cap:WE_{i}\to 2^{E}$.
w⊧e	Satisfaction, w⊧e⇔e∈cap(w).
ε	Runtime workflow executor assignment function, $\varepsilon :S⇀WE_{i}$; valid if (V1)–(V3).
$c_{z}\left(s\right)$	Consumer (choreographer or assigned executor) responsible for step *s* in session *z*.
$z\in Z_{i}$	Workflow session state, $z=(M,M_{A},\beta ,\varepsilon ,\xi ,status)$.
$Z_{i}$	Workflow session state space of $LC_{i}$.
*M*	Step-marking function, M:S→{inactive,enabled,running,completed}.
$M_{A}$	Action-marking function, $M_{A}:A\to \left\{\mathrm{inactive},\mathrm{active},\mathrm{completed}\right\}$.
β	Service-binding function, $\beta :S\to \Pi_{i}\cup \left\{⊥\right\}$.
ξ	Step-local execution-context function, $\xi :S⇀X_{i}$.
$X_{i}$	Domain of intermediate step-local execution data.
status	Overall workflow session status, status∈{running,completed,failed}.
$z\overset{\ell }{\to }z^{\prime }$	Labeled transition between workflow session states (Section 5.9).
ρ	Session parameters, ρ∈P, fixed at session creation.
asg(z,s)	Set of states after assigning a workflow executor to step *s*.
*D*	Application-specific data domain.
input	Input-construction function, $input:S\times Z_{i}⇀D$, $input\left(s,z\right)=\kappa_{s}\left(\rho ,\xi_{z}{|}_{dep(s)}\right)$, defined iff $\xi_{z}\left(p\right)$ is defined for all p∈dep(s).
$d_{s,z}$	Concrete input constructed for step *s* in session *z*, $d_{s,z}=input\left(s,z\right)$.
$compile_{i}$	Request-compilation function, $compile_{i}:S\times Z_{i}\to Req_{i}$.
$req_{s,z}$	Arrowhead request compiled from step *s* in session *z*, $req_{s,z}=compile_{i}\left(s,z\right)$.
λ	Session-dependent request-parameter function, $\lambda :S\times Z_{i}\to \Lambda_{i}$.
$\Lambda_{i}$	Domain of session-dependent request parameters and orchestration preferences.
$\pi_{s,z}\in \Pi_{i}$	Orchestration plan (service binding) selected for step *s* in session *z*.
$consume_{i}$	Service-invocation function, $consume_{i}:\Pi_{i}\times D\to D\cup \left\{\mathrm{fail}\right\}$.
$d_{s,z}^{\prime }$	Result returned by the service provider for step *s* in session *z*.
fail	Service-invocation failure symbol.

**Table 5:** Guarantees of the model, their formal basis, and where this paper demonstrates them.

Guarantee	Formal Basis	Practical Consequence	Demonstrated in
(a) Static validation	(W1)–(W4)	Faulty recipes are rejected before execution	Recipe definitions of Section 8
(b) No step runs on missing data	Lemma 1(iv), Lemma 2	A valid measurement exists before evaluation or actuation	Table 7 and Table 8; $s_{\mathrm{prog}}\to s_{\mathrm{chk}}$ in Section 8
(c) Termination, no deadlock, exact length	Theorems 1 and 2	Progress monitoring and log check (not a timeout)	11 transitions in Examples 1 and 2; 33 in Section 8
(d) Exclusive use of a shared resource	Proposition 1	The nutrunner is never used by two lines at once	Table 12

**Table 6:** Realization of the model elements in the kits for the workflow choreographer of Eclipse Arrowhead 4.6.2.

Model Element	Realization in the Kits
Plan *P*, Actions, $\alpha_{0}$, $next_{A}$	Plan request (ChoreographerPlanRequestDTO) with actions, firstActionName and nextActionName
steps, $next_{\alpha }$, $F_{\alpha }$	Steps of each action with nextStepNames and firstStepNames
θ(s), ι(s)	serviceRequirement: service definition and interface requirements
qnty(s)	quantity, equal to 1
Step identifier, dep(s)	staticParameters of the step (step, dep)
Consumer $c_{z}\left(s\right)$, η, ε	Every step runs through a registered choreographer executor. The choreographer asks the executor for its dependencies, orchestrates the service and the dependencies with the executor as requester, and starts the executor. For η(s) undefined, the executor stands for $CH_{i}$ (see the text below the table).
ρ, ξ, $\kappa_{s}$	A registered session-context service. The executor reads ρ and ξ restricted to dep(s) (a missing result is refused, cf. Lemma 2), composes the input, and stores ξ(s)
session creation, $z_{0}$	The line controller or clock starts the plan through the client service choreographer-start-session and binds ρ to the new session
S-Done, S-FailInvoke	The executor reports the step as done (SUCCESS) or failed (FATAL_ERROR) to the choreographer

**Table 9:** Application systems of the shared-tool example.

Application System	Services Provided	Role
$AS_{\mathrm{line},k}$, k=1,2,3	—	Line controller of $L_{k}$; starts a session.
$AS_{\mathrm{crib}}$	ReserveTool, ReleaseTool	Tool manager of the shared tool.
$AS_{\mathrm{mes}}$	GetProgram	Manufacturing execution system.
$AS_{\mathrm{agv}}$	TransportTool	AGV fleet manager.
$AS_{\mathrm{nr}}$	ConfigureTool, TightenFasteners	Controller of the nutrunner.
$AS_{\mathrm{qms}}$	EvaluateTightening, RecordTightening	Quality and traceability system.

**Table 10:** Requirements, delegation and data composition of the steps of the shared-tool recipe.

*s*	θ(s)	η(s)	dep(s)	$\kappa_{s}\left(\rho ,r\right)$
$s_{\mathrm{res}}$	ReserveTool	undef.	⌀	$\left(\rho_{\mathrm{tool}},\rho_{\mathrm{line}}\right)$
$s_{\mathrm{prog}}$	GetProgram	undef.	⌀	$\rho_{\mathrm{var}}$
$s_{\mathrm{move}}$	TransportTool	$e_{\mathrm{agv}}$	$\left\{s_{\mathrm{res}}\right\}$	$\left(\rho_{\mathrm{tool}},\rho_{\mathrm{crib}},\rho_{\mathrm{stn}},r\left(s_{\mathrm{res}}\right)\right)$
$s_{\mathrm{cfg}}$	ConfigureTool	undef.	$\left\{s_{\mathrm{prog}}\right\}$	$\left(\rho_{\mathrm{tool}},r\left(s_{\mathrm{prog}}\right)\right)$
$s_{\mathrm{tight}}$	TightenFasteners	undef.	$\left\{s_{\mathrm{cfg}}\right\}$	$\left(\rho_{\mathrm{tool}},r\left(s_{\mathrm{cfg}}\right)\right)$
$s_{\mathrm{chk}}$	EvaluateTightening	undef.	$\left\{s_{\mathrm{tight}},s_{\mathrm{prog}}\right\}$	$\left(r\left(s_{\mathrm{tight}}\right),r\left(s_{\mathrm{prog}}\right)\right)$
$s_{\mathrm{rec}}$	RecordTightening	undef.	$\left\{s_{\mathrm{tight}}\right\}$	$\left(\rho_{\mathrm{line}},\rho_{\mathrm{var}},\rho_{\mathrm{tool}},r\left(s_{\mathrm{tight}}\right)\right)$
$s_{\mathrm{ret}}$	TransportTool	$e_{\mathrm{agv}}$	⌀	$\left(\rho_{\mathrm{tool}},\rho_{\mathrm{stn}},\rho_{\mathrm{crib}}\right)$
$s_{\mathrm{rel}}$	ReleaseTool	undef.	$\left\{s_{\mathrm{res}}\right\}$	$\left(\rho_{\mathrm{tool}},r\left(s_{\mathrm{res}}\right)\right)$

**Table 11:** One failure-free run of the session of line $L_{2}$. The last column is the cumulative number of transitions. Parallel steps may interleave in any order; rule instances are written as rule(·) with the step abbreviated.

#	Transitions	Effect (Illustrative Values)	Σ
1	enable(res), enable(prog)	Both steps of $F_{\mathrm{prov}}$ become enabled.	2
2	bind, done for res and prog	$\xi \left(s_{\mathrm{res}}\right)=r_{2}$ (reservation token); $\xi \left(s_{\mathrm{prog}}\right)=\left(8,210\;N\cdot m,5\%\right)$.	6
3	enable, bind, done for move	Executor $w_{\mathrm{agv}}$ is assigned; $M(s_{\mathrm{move}})=\mathrm{running}$ during the AGV mission; $\xi (s_{\mathrm{move}})=$ arrival at ${\mathrm{st}}_{2}$.	9
4	enable, bind, done for cfg	Enabled only after move and prog completed; $\xi (s_{\mathrm{cfg}})=$ program loaded.	12
5	finish($\alpha_{\mathrm{prov}}$), start($\alpha_{\mathrm{use}}$)	Action marking migrates active→completed; next action becomes active.	14
6	enable, bind, done for tight	$\xi \left(s_{\mathrm{tight}}\right)=R=\left(T_{1},\ldots ,T_{8}\right)$.	17
7	enable, bind, done for chk and rec (parallel)	$\xi (s_{\mathrm{chk}})=\mathrm{OK}$; $\xi (s_{\mathrm{rec}})=$ stored.	23
8	finish($\alpha_{\mathrm{use}}$), start($\alpha_{\mathrm{ret}}$)	Return action becomes active.	25
9	enable, bind, done for ret	Executor assigned again; tool back in the crib.	28
10	enable, bind, done for rel	ReleaseTool processed with the token $\xi (s_{\mathrm{res}})$.	31
11	finish($\alpha_{\mathrm{ret}}$), close	status=completed.	33

## Data Availability

Interactive simulations of the two worked examples, and runnable kits for the Eclipse Arrowhead workflow choreographer (release 4.6.2), are available at https://arrowhead-workflows.vercel.app/ (accessed on 2 November 2026). The source code is available at https://github.com/palvarga/arrowhead-workflows (accessed on 2 November 2026) under the EPL-2.0 license.

## Abbreviations

The following abbreviations are used in this manuscript:

**Symbol**	**Definition**
⊧	Satisfaction relation between a workflow executor and a capability description, w⊧e⇔e∈cap(w).
∧	Logical conjunction.
∨	Logical disjunction.
¬	Logical negation.
⇒	Logical implication.
∀	Universal quantification.
∃	Existential quantification.
$\overset{\ell }{\to }$	Labeled state-transition relation of workflow sessions (Section 5.9).
$\overset{\cdot }{\to }$	Informal transformation notation used in the execution pipeline.
⇀	Partial-function notation.
×	Cartesian product.
∈	Set membership.
∉	Non-membership in a set.
⊆	Subset relation.
∪	Set union.
∩	Set intersection.
∖	Set difference.
⌀	Empty set.
|X|	Cardinality of set X.
N	Set of natural numbers.
$N_{0}$	Set of non-negative integers.
R	Set of real numbers.
